# Just-In-Time Adaptive Interventions for Weight Management Among Adults With Excess Body Weight: Scoping Review

**DOI:** 10.2196/76625

**Published:** 2025-12-25

**Authors:** Jia Ying Jennell Koh, Sherman Xuanming Tan, Xue Min Tan, Celine Yu Han Tan, Soo Ling Chan, James Wai Kit Lee, Amanda Yuan Ling Lim, Weiqin Lin, Han Shi Jocelyn Chew

**Affiliations:** 1 Alice Lee Centre for Nursing Studies, Yong Loo Lin School of Medicine National University of Singapore Singapore Singapore; 2 Division of Endocrinology Department of Medicine Ng Teng Fong General Hospital Singapore Singapore; 3 Department of Surgery National University Hospital Singapore Singapore; 4 Department of Endocrinology National University Hospital Singapore Singapore; 5 Department of Cardiology National University Heart Centre Singapore Singapore Singapore

**Keywords:** just-in-time adaptive intervention, obesity, weight loss, ecological momentary assessment, digital health

## Abstract

**Background:**

Just-in-time adaptive interventions (JITAIs) use real-time monitoring to deliver personalized support at optimal moments, demonstrating potential for improving lifestyle behaviors in weight management.

**Objective:**

This study provides an overview of how JITAIs have been used or developed for weight management in adults with excess body weight.

**Methods:**

This scoping review followed Arksey and O’Malley’s 5-step framework and the PRISMA-ScR (Preferred Reporting Items for Systematic Reviews and Meta-Analyses extension for Scoping Reviews) checklist to ensure methodological rigor. Eight electronic databases (PubMed, Cochrane Library, Embase, CINAHL, PsycINFO, IEEE Xplore, Scopus, and Web of Science) were searched from journal inception to November 13, 2024, along with gray literature and hand-searched references. Two independent reviewers conducted data extraction for all included studies. Descriptive statistics were used to summarize study characteristics, followed by a nonlinear, inductive qualitative content analysis of the extracted data to identify and synthesize recurring concepts and characteristics of JITAI-based weight management interventions.

**Results:**

Thirty-five studies on JITAIs for weight management were included, focusing on dietary behavior (25/35, 71.4%), physical activity (20/35, 57.1%), and self-weighing (17/35, 48.6%). Types of support included prompts (n=33), feedback (n=24), recommendations of coping strategies (n=7), and educational information (n=5). A total of 31.4% of studies used machine learning for decision-making, while the rest used rule-based algorithms. Retention rates varied from 74% to 100%, and compliance from 15.1% to 94.6%. Greater user engagement was associated with improved weight loss outcomes. Across interventions, significant improvements were observed in weight, waist circumference, BMI, and blood pressure, alongside increased physical activity, healthier dietary behaviors, and reductions in sedentary time.

**Conclusions:**

While JITAIs show potential for improving lifestyle habits by providing the right intervention at the right time and in the right setting, most studies lacked theoretical grounding and were not conceptualized as JITAIs. Furthermore, terminology and reporting were inconsistent, which hindered evaluation and comparison across studies. Nevertheless, most studies incorporated varied distal and proximal outcomes, behavioral theories, intervention delivery methods, and data acquisition methods, and demonstrated positive outcomes in weight, physical activity, and dietary behaviors. This review demonstrates JITAIs’ potential in weight management but highlights the field’s early stage of development. Future research should focus on improving reporting standards, optimizing JITAI components such as the integration of behavioral theories and machine learning, and enhancing user engagement and long-term effectiveness by incorporating passive sensing, personalization, and adaptive feedback mechanisms.

## Introduction

Overweight and obesity have reached epidemic levels that burden the public health care system by increasing the risk of complications such as diabetes mellitus, cardiovascular disease, hypertension, stroke, and some cancers [1]. According to the World Health Organization (WHO), the global prevalence of adult overweightness and obesity increased from 25% to 43% between 1990 and 2022 [2]. Its estimated economic burden in 2019 stood at US $1.33 trillion in high-income countries and US $3.19 billion in low-income countries [3]. The major challenge with weight management lies in the complex interplay of genetic, epigenetic, physiological, environmental, behavioral, and sociocultural factors [4]. Weight management approaches, such as bariatric surgery and pharmacological interventions, offer promising results; yet, sustainable and widely accessible weight management continues to rely fundamentally on lifestyle interventions [5]. Innovative solutions targeting lifestyle interventions are essential to improve weight loss and public health outcomes.

Just-in-time adaptive interventions (JITAIs) are an innovative digital behavior change intervention (DBCI) design that leverages real-time user data to deliver personalized support at moments of heightened vulnerability or receptivity, thereby enhancing behavior change [6-10]. Nahum-Shani et al [6,7], a pioneer in coining JITAI, identified 6 core design elements namely (1) proximal outcomes: short-term goals (eg, behavior change), (2) distal outcomes: long-term goals (eg, weight management), (3) decision rule: determining which intervention to deliver, (4) decision points: specific times when rules are applied, (5) intervention options: array of potential supports, and (6) tailoring variables: user data on internal states (eg, mood and motivation) and context (eg, schedule and environment). These elements work in concert to create a dynamic, responsive intervention system tailored to individual needs and circumstances.

The core mechanism of JITAIs is based on “if-then” decision points, in which the intervention is delivered only once specific criteria have been met. Nahum-Shani and Murphy [11] identified 3 elements of this decision point: the tailoring variables, the thresholds applied to the tailoring variable, and the intervention options. For example, a JITAI that tackles overeating can be operationalized based on the “if-then” decision point. An individual may log their calorie intake at every meal and set their recommended daily calorie limit to the recommended amount. If the individual’s calorie count, the tailoring variable, exceeds this threshold. In that case, they will then receive a notification from an application to monitor their dietary habits, with the notification serving as the intervention option.

A tailoring variable refers to the factor an intervention seeks to target, with data collected using repeated sampling methods (ie, active, passive, or both) to obtain temporally dense data on personal characteristics [9]. Active assessment often includes self-reports, including ecological momentary assessments (EMAs) and other user-initiated reports that rely on the JITAI framework [6] to provide adaptive support. Passive assessment, conversely, involves data collection through sensors (eg, smartwatches and smartphones) and digital sources (eg, digital calendars and web browsing history) with minimal user involvement [6,8,9].

The subsequent threshold of the tailoring variable then determines whether the intervention is necessary, informed by existing literature and adjusted to participant-specific contexts. An effective threshold should identify states of vulnerability (eg, high-risk moments for lapse), states of opportunity (eg, opportunities to persuade the user to walk during commuting), and user receptivity [7], which the JITAI could address in real time.

Finally, JITAIs deliver tailored interventions, adjusting the content, dose, and timing based on the user’s social context to increase the likelihood of adaptation of the JITAI intervention [7]. This dynamic design makes JITAIs well-suited to address the multifaceted and fluid nature of weight management, potentially overcoming the limitations of traditional weight management programs. JITAIs have demonstrated versatility beyond weight management, being applied to various health behavioral issues such as alcoholism, smoking cessation, mental illness, and physical inactivity [8].

While JITAIs hold strong potential for weight management [10], their development is still in its infancy. Studies lack consistency and standardization in the application of the 6 core components of JITAIs [8]. Moreover, research design aligning with JITAIs’ definitions has used various terms, such as “dynamic tailoring,” “dynamically and individually tailored ecological momentary interventions (EMIs),” “intelligent real-time therapy,” and “adaptive context-aware interventions” [6,12,13].

To the best of our knowledge, few reviews on JITAIs exist, and none focus specifically on their applicability to weight management or dietary behavior. The limited relevant reviews include a systematic review by Hardeman et al [14] on JITAIs for physical activity, a meta-analysis by Wang and Miller [12] covering JITAIs targeting various behavioral issues, and 2 scoping reviews evaluating intervention reporting quality and automation standards in JITAIs studies [15,16]. However, these studies scarcely addressed or did not mention weight management and the potential role of JITAIs. Given the scarcity and heterogeneity of weight management JITAI studies, as well as the complexity of JITAI designs, a scoping review was conducted to contextualize existing knowledge, provide insights into complex and heterogeneous literature, identify gaps, and advance the field [17,18]. This paper aims to provide an overview of how JITAIs could be used for weight management among adults with excess body weight.

## Methods

### Guidance Framework

This scoping review was conducted following Arksey and O’Malley’s 5-step framework: (1) identifying the research question, (2) identifying relevant studies, (3) study selection, (4) charting the data, and (5) collating, summarizing, and reporting the results [19]. The PRISMA-ScR (Preferred Reporting Items for Systematic Reviews and Meta-Analyses extension for Scoping Reviews) checklist [20] was used to ensure methodological rigor (Table S1 in Multimedia Appendix 1).

### Identifying the Research Questions

The review aimed to address the following questions:

1. What conceptual designs do current weight management JITAIs adopt?
2. What are the features of current weight management JITAIs?
3. What is the effectiveness of current weight management JITAIs in improving user-related outcomes?

### Identifying Relevant Studies

The International Prospective Register of Systematic Reviews (PROSPERO) database was first searched to ensure no duplicate reviews had been published. A 3-step comprehensive search strategy was developed in consultation with an academic librarian and conducted by XMT. First, a preliminary search was conducted on PubMed using the search string (“just-in-time adaptive interventions” AND “weight management”) to identify potential keywords. Second, 8 electronic databases (PubMed, Cochrane Library, Embase, CINAHL, PsycINFO, IEEE Xplore, Scopus, and Web of Science) were searched from journal inception until November 13, 2024. The final keywords used were “just-in-time adaptive intervention*,” “just-in-time,” “ecological momentary intervention*,” “context-aware*,” “adaptive NEAR/4 intervention*,” “digital behavio*r change,” “dynamic* NEAR/4 tailor*,” “real-time NEAR/4 tailoring,” “real-time therap*,” “tailor* NEAR/4 feedback,” “adapt* NEAR/4 feedback,” “real-time NEAR/4 feedback,” “microrandomi*,” “overweight,” “obes*,” “weight loss,” “weight management,” “weight reduction,” “body weight,” “BMI,” and “body mass.” The comprehensive search strategy for all databases is provided in Table S2 in Multimedia Appendix 2. To reduce publication bias and increase comprehensiveness, 2 gray literature databases (Science.gov and ProQuest Dissertations and Theses Global) and the first 200 results retrieved from Google Scholar [21] were also searched. In addition, 3 trial registries (ClinicalTrials.gov, ISRCTN registry, and WHO International Clinical Trials Registry Platform) were searched for ongoing studies. Finally, backward citation searching of all related reviews, frameworks, and papers included in the title and abstract screening was also conducted.

### Study Selection

All studies were imported into EndNote (version 21; The EndNote Team), where duplicates were removed using the EndNote automation tool [22]. Eligibility criteria were developed according to the population-concept-context framework (Table S3 in Multimedia Appendix 2 [20]). Papers were included if they described JITAIs for weight loss in adults (aged ≥18 years) with excess body weight (BMI ≥25). All English-language study designs and publication types (developmental papers, conceptual papers, feasibility studies, secondary analyses, theses, conference proceedings, and journal papers) were included to broaden the scope of the search, excluding reviews and frameworks. Studies that did not use the term “JITAI” were determined to be JITAIs if they met specific criteria, including automated devices, real-time monitoring, automatic data processing, adaptive provision of support (content, dose, and timing), and system-delivered intervention delivery. Studies were excluded if they lacked sufficient information to determine whether they were JITAIs or lacked descriptions of their functions, mechanisms, or application in weight management. Study screening was conducted by XMT, and citations were managed using EndNote (version 21) software [22].

Study screening and selection were conducted by a single reviewer (XMT) due to resource limitations. While independent screening by 2 reviewers is recommended to minimize bias and enhance comprehensiveness, a single-reviewer approach was implemented due to practical and logistical constraints inherent to the project timeline. To ensure methodological rigor, explicit inclusion and exclusion criteria were developed a priori and reviewed by a second reviewer (HSJC). These criteria were applied systematically across all databases, guided by a standardized screening protocol. All decisions were comprehensively documented to ensure transparency, consistency, and reproducibility of the selection process.

### Charting the Data

Data extraction using Google Sheets was first pilot tested by XMT on 5 randomly selected papers and reviewed with HSJC to ensure consistency and clarity of data fields. The data extraction fields were iteratively refined through discussion until consensus was reached between the 2 reviewers (XMT and HSJC). The final data extraction framework included author, year, country, publication type, study design, duration of intervention, sample size, intervention used, compliance rate, mode of delivery, proximal outcome, behavioral theory used, wearables, algorithm used, intervention description, and data acquisition. Two reviewers (XMT and JYJK) independently conducted data extraction for all included studies. Discrepancies were discussed and resolved by a third reviewer (HSJC).

### Collating, Summarizing, and Reporting the Results

Following the Joanna Briggs Institute scoping review guidance [23], both quantitative and qualitative methods were used to collate and summarize the data. Descriptive statistics were performed by XMT using Microsoft Excel [24] to summarize study characteristics in terms of frequencies, percentages, and ranges, and were reviewed by JYJK to ensure accuracy and consistency. A nonlinear, inductive qualitative content analysis was then conducted to identify and synthesize recurring concepts, characteristics, and components of JITAI-based weight management interventions. This approach was chosen due to the limited evidence on this topic and its suitability for clarifying the key characteristics and components of weight management JITAIs [18]. The qualitative content analysis followed 6 iterative stages: (1) immersion in data, (2) inductive extraction and analysis, (3) open coding, (4) developing the coding framework, (5) extraction and organization, and (6) categorization [23]. Stages 1 and 2 (immersion and inductive analysis) were conducted independently by XMT and JYJK to ensure an unbiased interpretation of the data. Open coding was then performed collaboratively through iterative discussions between XMT and JYJK to develop the preliminary coding framework, with discrepancies resolved by HSJC. The finalized coding framework was subsequently applied across all included studies, during which extracted data were organized and categorized through an iterative comparison process to refine the concepts, characteristics, and components of weight management JITAIs. No critical appraisal of methodological quality was conducted, consistent with the Joanna Briggs Institute scoping review guidance [18], as the objective of this review was to provide an overview of existing evidence regardless of methodological quality [20].

## Results

### Database Search

**Figure 1:**
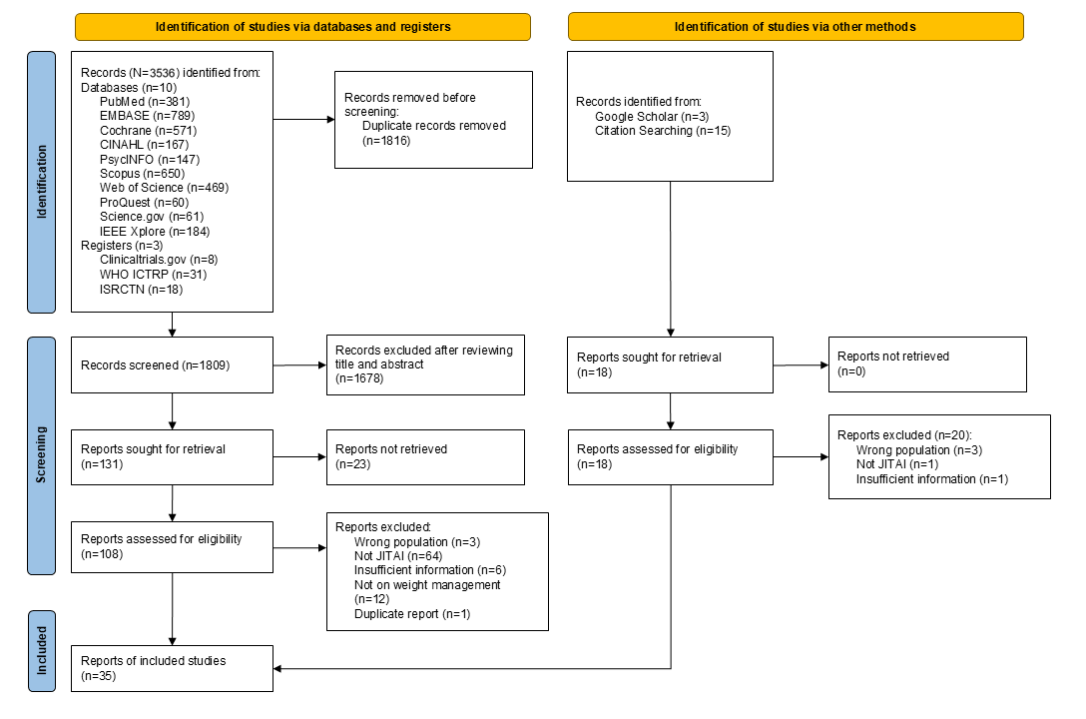
PRISMA (Preferred Reporting Items for Systematic reviews and Meta-Analyses) flow diagram. ISRCTN: International Standard Randomized Controlled Trial Number; JITAI: Just-In-Time Adaptive Intervention; WHO ICTRP: World Health Organization International Clinical Trials Registry Platform.

The database and register search yielded 3625 records. After removing 1816 duplicates with the EndNote automation tool [22], 1809 records underwent title and abstract screening. This process resulted in 131 reports, with full-text extraction available for 108 reports. In addition, 3 records from Google Scholar and 15 from citation searching were also retrieved. A total of 35 full texts were ultimately included in this review, along with 5 relevant ongoing trials identified. The PRISMA (Preferred Reporting Items for Systematic Reviews and Meta-Analyses) flow diagram with reasons for exclusions is provided in Figure 1.

### Characteristics of Included Studies and Participants

The 35 papers comprised peer-reviewed journal papers (n=25), theses (n=3), and conference proceedings (n=7). Study designs varied, including developmental studies (n=5), mixed method studies (n=2), experimental studies (n=20), secondary analyses (n=7), and a qualitative study (n=1). The majority of the reports originated from the United States (n=29), with others from the Netherlands (n=3), the United Kingdom (n=1), Turkey (n=1), and India (n=1). Intervention durations ranged from 14 days [25] to 12 months [26-31]. Table 1 provides a summary of study characteristics, and Table 2 provides the individual study characteristics.

**Table 1:** Summary of study characteristics (N=35).

Study characteristic			Value, n (%)
**Publication type**			
	Journal paper	25 (71.4)	
	Conference proceeding	7 (20)	
	Thesis	3 (8.5)	
**Study design**			
	Developmental study	5 (14.3)	
	Mixed method study	2 (5.7)	
	Experimental study	20 (57.1)	
	Secondary analysis	7 (20)	
	Qualitative study	1 (2.9)	
**Country of origin**			
	United States	29 (85.7)	
	The Netherlands	3 (8.6)	
	United Kingdom	1 (2.9)	
	Turkey	1 (2.9)	
	India	1 (2.9)	
**Health behavior as proximal outcomes**			
	Dietary behavior	25 (71.4)	
	Dietary structure	13 (37.1)	
	Eating rate	4 (11.4)	
	Dietary lapses	8 (22.9)	
	Physical activity	20 (57.1)	
	Increase in physical activity	19 (54.3)	
	Decrease in sedentary behavior	3 (8.57)	
	Self-weighing	17 (48.6)	
**Behavioral theory adoption**			
	Reported adoption of at least 1 behavioral theory	14 (40)	
	Social cognitive theory	9 (25.7)	
	Kanfer’s theory of self-regulation	7 (20)	
	Fogg behavior model	2 (5.7)	
	Transtheoretical model	2 (5.7)	
	Health action process approach	1 (2.9)	
	None reported	21 (60)	
**Type of support**			
	Prompt and feedback	17 (48.6)	
	Prompt only	4 (11.4)	
	Feedback only	2 (5.71)	
	Prompt and recommendation of a coping strategy	7 (20)	
	Prompt, feedback, and educational information	5 (14.3)	
**Intervention delivery**			
	Application message	24 (68.6)	
	Text message	7 (20)	
	Audio feedback	3 (8.57)	
	Mobile phone haptic vibration	1 (2.9)	
	Augmented fork vibrotactile feedback	1 (2.9)	
**Employment of wearables**			
	At least 1 wearable used	16 (45.7)	
	None used	19 (54.3)	
**Data acquisition**			
	Both active and passive assessments	9 (25.7)	
	Passive assessment only	12 (34.3)	
	Active assessment only	14 (40)	
**Active assessments: tailoring variables measured**			
	Ecological momentary assessment	8 (22.9)	
	Self-reported questionnaires	15 (42.9)	
	Self-reported weight measurements	15 (42.9)	
**Passive assessments: sensors and tailoring variables measured**			
	Accelerometer	19 (54.3)	
	Physical activity	18 (51.4)	
	Eating rate	1 (2.9)	
	Time	10 (28.6)	
	Location sensor	4 (11.4)	
	Galvanic sensor	2 (5.7)	
	Thermal flow sensor	2 (5.7)	
	Skin temperature sensor	2 (5.7)	
	Air temperature sensor	2 (5.7)	
	Image capturing monitor (captures dietary structure)	2 (5.7)	
	Previous user responses	3 (8.6)	
	Digital weighing device	2 (5.7)	
	Weather	2 (5.7)	
	Personal calendar	2 (5.7)	
	Sound sensor (dietary structure and detection of eating episodes)	1 (2.9)	
**Decision-making algorithm** us**ed**			
	Rule-based algorithm	24 (68.6)	
	Machine learning	11 (31.4)	

**Table 2:** Individual study characteristics (n=35).

Author; Year	Country; publication type	Study design; duration; n	Intervention used	Compliance	Key outcomes
Goldstein, 2016 [32]	United States; thesis dissertation	Pilot trial; 6 weeks; n=12	DietAlert and WW^a^	EMA^b^ survey response: 94.6%; retention: 100% (with compensation)	Model accuracy: 0.67-0.72; specificity: 0.68-0.72; sensitivity: 0.45-0.70; higher data volume ↑ model outcomes, especially sensitivity.
Goldstein et al, 2017 [10]	United States; journal paper	Developmental; N/A^c^; N/A	DietAlert and WW	N/A	N/A
Forman et al, 2019a [33]	United States; journal paper	Pilot; 8 weeks; n=43	OnTrack (formerly DietAlert) and WW	EMA survey response: 85.1%; retention: 97.7%	Negative predictive value 80%; 70.15% alerts opened; 3.13% weight loss; app easy to use, had minimal issues, moderately useful and enjoyable; unplanned lapses ↓ over time.
Forman et al, 2019b [34]	United States; journal paper	RCT^d^; 10 weeks; n=181	OnTrack (formerly DietAlert) and WW	EMA survey response: 62.9%; retention: 88.4%	Specificity 83.8%; Sensitivity 69.2%; 46.9% alerts opened; 2.1% weight loss when moderated by diet type; high satisfaction reported; 72.8% risk alerts received as helpful or accurate; dietary lapses ↓ over time.
Goldstein et al, 2020 (Primary) [35]	United States; journal paper	Randomized trial; 10 weeks; n=121	OnTrack (formerly DietAlert) and WW	EMA survey response: 65.4% (8 questions) and 60.5% (17 questions); retention: 84.3%	Accuracy 79.7% (8 questions) vs 79.9% (17 questions); specificity 84.4% (8 questions) vs 81.7% (17 questions); sensitivity 71.6% (8 questions) vs 77.7% (17 questions); 46.9% alerts opened; 3.4% weight loss; 72.84% risk alerts received as helpful or accurate.
Goldstein et al, 2021a (Secondary) [36]	United States; journal paper	Secondary analysis of randomized trial; 10 weeks; n=121	OnTrack (formerly DietAlert) and WW	EMA survey response: 63.1%	0.49% weight loss; 50.3% accessed intervention library per week; lapse frequency not statistically significantly associated with percentage weight loss.
Goldstein et al, 2021b (Secondary) [37]	United States; journal paper	Secondary analysis of randomized trial; 10 weeks; n=121	OnTrack (formerly DietAlert) and WW	EMA survey response: 62.9%	Lapses occurrence: multiple 28.5% (LASSO accuracy 69.8%), planned 22.9% (67.2%), off-plan 16.4% (67.9%), larger portion 12.5% (67.7%), unknown points 10.8% (70.6%), unintended time 8.1% (69%); ↓unplanned lapses (larger portion, unintended time, multiple) ↑ weight loss; 3.7% weight loss observation.
Burke et al, 2017 [29]	United States; journal paper	Pilot randomized trial; 12 weeks; n=39	SMARTER and LoseIt!	SM^e^: 53.5%; SM and FB^f^: 55.9%; SM, FB, and F2F^g^: 65.3% adherent to self-monitoring; retention was 74% (with compensation).	All groups lost weight and ↓SBP^h^; no between-group differences for weight loss, SBP, DBP^i^, and self-efficacy; DBP ↓ in SM and SM+FB+F2F; ↑ self-efficacy only in SM.
Burke et al, 2020 [27]	United States; journal paper	Developmental; N/A^c^; N/A	SMARTER and Fitbit	N/A	N/A
Burke et al, 2022 [28]	United States; journal paper	RCT; 6 months; n=502	SMARTER, Fitbit, and 1-to-1 90-minute dietary counselling	Around 54.8% of feedback messages were opened; retention was 86%.	Both groups lost weight, ↓ BMI, and WC^j^; no between-group differences.
Burke et al, 2022 (Primary) [26]	United States; journal paper	RCT; 12 months; n=502	SMARTER, Fitbit, and 1-to-1 90-minute dietary counselling	Around 42.2% of feedback messages were opened; retention was 78.5%.	Both groups lost significant weight (–2.16 kg); no between-group weight loss difference; % days adherent to the calorie goal was higher and declined more slowly in SM and FB; ↑ feedback messages opened→↑ adherence to calorie goal and weight loss.
Cheng et al, 2023 (Secondary) [30]	United States; journal paper	Secondary analysis of RCT; 12 months; n=502	SMARTER, Fitbit, and 1-to-1 90-minute dietary counselling	Not stated	Minimal diet quality improvement overall; weight loss ≥5% linked to higher HEI-2015^k^ [38] scores at 6 months, but not sustained at 12 months.
Bizhanova et al, 2022 (Secondary) [39]	United States; journal paper	Secondary analysis of RCT; 12 months; n=502	SMARTER, Fitbit, and 1-to-1 90-minute dietary counselling	Around 66.5% met the PA^l^ goal at week 1; median adherence was higher in SM and FB (165.2%) vs SM (106.3%) at 12 months, but no within-group differences; adherence was nonlinear and nonsignificant in both groups.	Greater PA adherence was linked to male sex, more feedback message engagement, higher baseline self-efficacy, week-1 adherence, greater weight loss at week 4 and 12 months, and fewer mental health issues; ML^m^ models (random forest regression, regression tree model, and LASSO^n^ model) identified week-1 PA attainment as the strongest predictor.
Kariuki et al, 2024 [40]	United States; journal paper	Qualitative study; 6 focus groups; n=23	N/A	N/A	Successful weight loss group: SMARTER helpful, organized, effective with consistent use; Unsuccessful weight loss group: weight gain or no weight loss experienced, feedback discouraging, harsh or out of context; Overall: both diet and PA are key in weight loss; most messages are inaccessible due to a 1-hour window, timing, and personalization needed for such applications.
Bond et al, 2014 (Primary) [41]	United States; journal paper	Quasi study; 4 weeks; n=35	B-MOBILE	Not stated; 85.7% retention (with compensation).	↓ sedentary time, ↑ light, and MVPA^o^ in all conditions; 3-minute breaks gave the largest effects (–5.9% sedentary time, +3.9% light PA); 90% found real-time feedback motivating and found smartphone prompts helpful in reducing sedentary time; 6-minute PA breaks were most preferred.
Thomas and Bond, 2015 (Secondary) [42]	United States; journal paper	Secondary analysis of quasi-study; 4 weeks; n=35	B-MOBILE	Adherence: 3 minutes (89.4%), 6 minutes (86.7%), and 12 minutes (77%).	High engagement across all conditions; 3-minute prompts produced the most prompts/day (7), walking breaks/day (6.5), and shortest latency (23 minutes); walking minutes significantly different in 3-minutes (37.2) and 6-minutes (38.7) condition vs 12-minutes (32.5 minutes); 3-minute condition yielded similar total walking time as 6-minute as participants often exceeded required duration of prompt.
Westenenk, 2023 [25]	The Netherlands; thesis dissertation	Microrandomized trial; 14 days; n=13	Ancora Health	92.9% retention	Sending 1 prompt ↑ steps vs no prompt; evening prompts ↓ steps vs morning; 2 prompts/day ineffective.
Shapiro et al, 2012 [31]	United States; journal paper	RCT; 12 months; n=170	Text4Diet	Increased adherence to knowledge testing, decreased adherence to first and follow-up weight and step queries over time, and 76% retention (with compensation).	Modest weight loss, no group differences; higher SMS text messaging adherence → greater weight loss; ↑ steps linked with ↑ weight loss at 12 months; moderately strong satisfaction with program; ↑ satisfaction with pedometer component → ↑ weight loss at 6 months; 85% would pay US $4.99/month.
Haggerty et al, 2016 [43]	United States; journal paper	Randomized trial; 6 months; n=20	Text4Diet	100% retention	90% lost weight; telemedicine > texting (–7.6% vs –4.1%); ↓ interleukin-2 (IL-2) postintervention.
Gupta and Sood, 2015 [44]	India; conference paper	RCT; 4 weeks; n=33	Let’s Exercise	Not stated	App rated effective and useful; 84.6% satisfied; 60% would continue using the app.
Patrick et al, 2009 (Primary) [45]	United States; journal paper	RCT; 4 months; n=65	mDIET	Two-thirds of messages were responded to by participants at the end of 4 months, and retention was 83.3%.	Intervention lost –3.16% weight vs –1.01% control; satisfaction high (92% would recommend).
Norman et al, 2012 (Secondary) [46]	United States; journal paper	Secondary analysis of RCT; 4 months; n=65	mDIET	Not stated	Intervention improved weight change, fruit and vegetable intake, and eating behavior; fruit and vegetable intake and eating behavior were inversely associated with weight loss and were mediators of weight loss.
Valle, et al, 2020 (Primary) [47]	United States; journal paper	Microrandomized trial; 12 weeks; n=53	Nudge	Two-thirds of messages were viewed by participants, and retention was 98.1%.	Message viewing declined weekly (–0.15/day) and with weight gain (+1 lb = –0.08) or longer lapses in weighing (–0.063/day). Viewing also fell with activity tracking lapses (–0.03/day). Likelihood of message viewing increased with more previous messages viewed (+0.07 per 1%) and more days meeting diet goals (+0.14/day).
Hurley et al., 2024 (Secondary) [48]	United States; Journal Paper	Secondary analysis of microrandomized trial; 12 weeks; n=53	Nudge	Not stated	Each unmet goal decreased the odds of viewing messages by 34.8%. Odds of viewing also declined daily (OR=0.977). Baseline depressive symptoms did not moderate these effects.
Rajanna et al, 2014 [49]	United States; conference paper	Developmental; not stated; user study (n=4), formative evaluation (n=2), summative evaluation (n=2)	Step Up Life	Not stated	Around 80% of users across the 3 evaluation phases (user study, formative evaluation, and summative evaluation) showed strong interest in the app.
Van Beurden et al, 2021 [50]	United Kingdom; journal paper	Developmental; N/A; N/A	ImpulsePal	N/A	N/A
Chen et al, 2024 [51]	United States; conference paper		GatorTrack and FatSecret	Completed 57.14% of daily logs, 15.09% of notification clicks, and 18% of notifications; 100% retention (with compensation).	Context-based notifications led to shorter click response time (12.33 vs 18.42 minutes), higher click rate (19.05% vs 13.96%), and higher completion rate (21.77% vs 17.32%). The average overall log rate was higher in the context-based condition (58.87% vs 55.54%) but was not significant.
Everett et al, 2018 [52]	United States, journal paper	Quasi-experimental (1-group pretest-posttest); 3 months; n=55	Sweetch	86% retention	↑ PA (2.8 metabolic equivalent task h/wk), ↓ weight (–1.6 kg, 2%), ↓ BMI (–0.6 kg/m2), ↓ WC (–1.4 cm), and ↓ A1c (–0.1%); high acceptability (78%).
Purpura et al, 2011 [53]	United States; conference paper	Randomized trial (conceptual); 9 weeks; n=26	Fit4Life (fictional)	N/A	Focuses on ethical reflection rather than efficacy.
Spanakis et al, 2017 [54]	The Netherlands; journal paper	Mixed method (study I: developmental and 2 weeks; study II: RCT and 8 weeks); n=100	ThinkSlim	Study I: 80%-81% of assessments done, study II: 70.5% of assessments done, and ~9.9 hours of application usage.	ML clustered 6 eater types; study II showed the feasibility of adaptive feedback based on these 6 eater types.
Finkelstein et al, 2015 [55]	United States; conference paper	Pilot, randomized crossover study; 8 weeks; n=30	Fitbit One and Android smartphone app	90% retention	Inactivity decreased during “message-on” periods (24.6% vs 30.4%), and step count increased (6199 vs 5615 steps). Most participants expressed high acceptance and willingness to use the app in the future.
Hermsen et al, 2019 [56]	The Netherlands; journal paper	RCT; 15 weeks; n=141	10sFork	86.5% retention (with compensation). In the group using the online dashboard, only 55.3% used the dashboard.	Eating rate slowed (−1.8 bites/min), the success ratio of >10 seconds between bites increased by 22.5%, and BMI decreased by 0.5-0.8; the dashboard added no benefit. Vibrotactile feedback had a small to moderate effect on bite rate and a moderate to large effect on the success ratio, and both effects remained significant for 8 weeks.
Mendi et al, 2013 [57]	Turkey; conference paper	Developmental; N/A; N/A	Android app and wrist-worn sensor	N/A	N/A
Moses et al, 2023 [58]	United States; conference paper	Mixed method (pilot quantitative study and qualitative study); not stated; n=29	SMS JITAI^p^	Not stated	Participants preferred non-numeric messages (nonsignificant), although numeric messages were still positively received. There was a statistical significance between message type and comprehension error rates.
Gao, 2021 [59]	United States; thesis dissertation	Quasi (1-group posttest); not stated; lab testing (n=28) and field testing (n=4)	ADM-IPA^q^	Not stated	More than 43% of segments were detected as eating activities, with the best accuracy of 74.3%.

^a^WW: Weight Watchers.

^b^EMA: ecological momentary assessment.

^c^N/A: Not applicable.

^d^RCT: randomized controlled trial.

^e^SM: self-monitoring.

^f^FB: feedback.

^g^F2F: face-to-face.

^h^SBP: systolic blood pressure.

^i^DBP: diastolic blood pressure.

^j^WC: waist circumference.

^k^HEI-2015: Healthy Eating Index 2015.

^l^PA: physical activity.

^m^ML: machine learning.

^n^LASSO: least absolute shrinkage and selection operator.

^o^MVPA: moderate to vigorous physical activity.

^p^JITAI: just-in-time adaptive intervention.

^q^ADM-IPA: Automated Diet Monitoring Intelligent Personal Assistant.

The review identified 19 distinct JITAI interventions from the 35 included papers: the SMARTER Trial [26-30,39,40], B-MOBILE [41,42], DietAlert [10,32-37], Let’s Exercise [44], mDIET [45,46], Text4Diet [31,43], Nudge [47,48], Step Up Life [49], ImpulsePal [50], GatorTrack [51], Sweetch [52], Fit4Life [53], ThinkSlim [54], Ancora Health [25], 10sFork [56], Automated Diet Monitoring Intelligent Personal Assistant (ADM-IPA) [59], and 3 were unnamed [55,57,58]. Table 3 provides a summary of the intervention features.

Only 5 of the interventions, comprising 13 studies, explicitly used the intervention design terminology “JITAI” [10,25,32-37,41,42,47,48,58].

**Table 3:** Summary of intervention features (n=19).

Name of intervention	Delivery	Intervention	Proximal outcome	Theory	Algorithm
DietAlert [10,32-37]	Active (event- and time-based EMA^a^ prompts + user-initiated EMA)	App messages: coping strategies and prompts; event- and time-based EMA sent 6×/day on lapse triggers, risk alerts, and access to on-demand education library	Dietary lapses	Not stated	ML^b^; 2-week group data were used to train the model, enabling individual adaptation in real time
SMARTER [26-30,39,40]	Active (FitBit self-reporting diet and weight); passive (FitBit PA^c^)	App messages: prompt and feedback; 3 tailored PA/diet messages daily; weight feedback every 6-8 days; monthly rotation	Diet, PA, and weight SM^d^	Kanfer’s self-regulation theory and social cognitive theory	Rule-based
B-MOBILE [41,42]	Passive (smartphone and SenseWear Mini armband)	App messages: prompts for 3-, 6-, or 12-minute PA breaks after 30-120 minutes of sedentary behavior	Increased PA and decreased sedentary time	Not stated	Rule-based
Ancora Health [25]	Passive (smartphone or wearable)	App messages: prompt and feedback; step-goal feedback tailored to achievement, phase of change (intender or actor), and time of day	Step count	Health action process approach	Rule-based
Text4Diet (modified version of mDIET) [31,43]	Active (self-reported weight and step count; knowledge-based questions)	Text (SMS text messaging or MMS^e^): prompts, feedback, and educational information; 4×/day for 12 months	Step count and weight self-monitoring	Social cognitive theory	Rule-based
Let’s Exercise [44]	Passive (smartphone sensors: location, weather, and time)	App messages: prompts and feedback; motivational or context-based PA messages (round-robin selection)	PA	Not stated	Rule-based
mDIET [45,46]	Active (self-reported weight, knowledge-based, eating-behavior questions)	Text (SMS text messaging or MMS): prompts, feedback, and educational information; >3000 unique messages (half interactive) and weekly weight-change graphs	Weight self-monitoring	Not stated	Rule-based
Nudge [47,48]	Active (weight and diet); passive (PA via activity tracker)	App messages: prompts and feedback; daily prompts (max 1 per behavior/day) for self-monitoring, encouragement, and feedback	Weight self-monitoring, diet, and PA	Not stated	Rule-based microrandomization
Step Up Life [49]	Passive (smartphone accelerometer, age, location, time, weather, and calendar)	Mobile phone haptic vibrations: prompt and feedback on exercise suggestions	PA	Fogg Behavior Model	ML
ImpulsePal [50]	Active (emergency button—to be pressed when strong cravings are experienced); passive (location)	App messages: prompts at high-risk locations, when cravings occur, or during inactivity	Resisting temptations and cravings	Not stated	Rule-based
GatorTrack + FitBit [51]	Active (diet, PA, weight logs, self-ratings of weight-related variables); passive (PA transitions)	App messages: prompts; daily or weekly notifications based on self-monitoring data and PA transition	Diet, PA, and weight self-monitoring	Fogg Behavior Model	Rule-based
Sweetch [52]	Passive (calendar, location, digital phenotype, and weight scale)	App messages: feedback; personalized push notifications for PA, weight, and diet goals	PA, weight self-monitoring, and diet	Transtheoretical mode	ML
Fit4Life *(conceptual)* [53]	Passive (data recorder, earpiece, Thinsert, heart rate monitor, and metabolic lancet)	Audio feedback: prompts and feedback; audio guidance on diet and PA, social-support prompts, social media updates	BMI, diet, and PA	Not stated	Rule-based
ThinkSlim [54]	Active (EMA—random vs event sampling)	App messages: prompts and feedback; adaptive feedback via app notifications	Unhealthy eating events	Not stated	ML
Android app and Fitbit One [57]	Passive (step count)	Text (SMS text messaging): prompts, feedback, and educational information; tailored weekly exercise education links and daily step reports	Sedentary time	Not stated	Rule-based
10sFork [56]	Passive (fork sensors or actuators that provide real-time vibrotactile feedback on eating rate)	Vibrotactile feedback fork: feedback; the fork vibrates if bites are <10 seconds apart, and the online dashboard	Bite rate, success ratio (>10 seconds), and BMI	Not stated	Rule-based
Android app + Wrist-Worn Sensor [58]	Passive (bites taken from wrist accelerometer)	Audio feedback and SMS text messages: prompts and feedback; real-time bite count and rate feedback, alerts if >5 bites/min	Eating rate	Not stated	Rule-based
JITAI^f^ SMS text messages [60]	Passive (image-capturing sensors)	Text (SMS text messaging): prompts and feedback; energy intake and eating rate	Amount of food intake and speed	Not stated	Rule-based
Automated Diet Monitoring Intelligent Personal Assistant (ADM-IPA) [59]	Passive (chewing and swallowing sounds captured by Bluetooth headsets)	Audio feedback: prompts and feedback; automated eating detection (overeating, snacking, skipping meals, and irregular timing) with corrective prompts	Unhealthy eating frequency, timing, and portion size	Fogg Behavior Model and the transtheoretical model	ML

^a^EMA: ecological momentary assessment.

^b^ML: machine learning.

^c^PA: physical activity.

^d^SM: self-monitoring.

^e^MMS: multimedia messaging service.

^f^JITAI: just-in-time adaptive intervention.

### JITAI Conceptual Design

A more detailed description of the JITAI conceptual components is provided in Tables S4 and S5 in Multimedia Appendix 2.

#### Behavioral Theory Adoption

Only 14 studies reported adopting a theoretical base in designing their JITAIs. The most used theory was Social Cognitive Theory (n=9) [26-31,39,40,43], followed by Kanfer’s Theory of Self-Regulation (n=7) [26-30,39,40], Fogg Behavior Model (n=2) [49,51,59], Transtheoretical Model (n=2) [52,59], and Health Action Process Approach (n=1; Tables 1 and 3) [25]. Although Kanfer’s Theory of Self-Regulation was used in 7 different studies [26-30,39,40], they were all derived from the development and evaluation of the SMARTER application, compared to other theories, which were applied across multiple, distinct interventions.

#### Distal and Proximal Outcomes

All studies shared weight loss as the distal outcome. Weight loss measures included weight (n=8) [31,43,45-48,50,52], percent weight loss (n=12) [10,26-30,33-37,39,40,43,52], BMI (n=8) [10,33-37,52,56], and waist circumference (n=1) [52]. Proximal outcomes varied across dietary behavior (n=25), physical activity (n=20), and self-weighing (n=17). Dietary behaviors were further subcategorized into (1) dietary structure (eg, measurement of caloric intake and food types; n=13) [26-30,39,40,47,48,51-54], (2) eating rate (n=4) [56-59], and (3) dietary lapses (n=8) [10,32-37,50]. Physical activity was divided into (1) increases in physical activity (n=19) [25-31,39-44,47-49,51-53] and (2) decreases in sedentary behavior (n=3) [41,42,55]. Self-weighing was used to increase users’ weight consciousness (Tables 1 and 3) [26-31,39,40,43,45-48,51-53,56].

### JITAI Features

**Figure 2:**
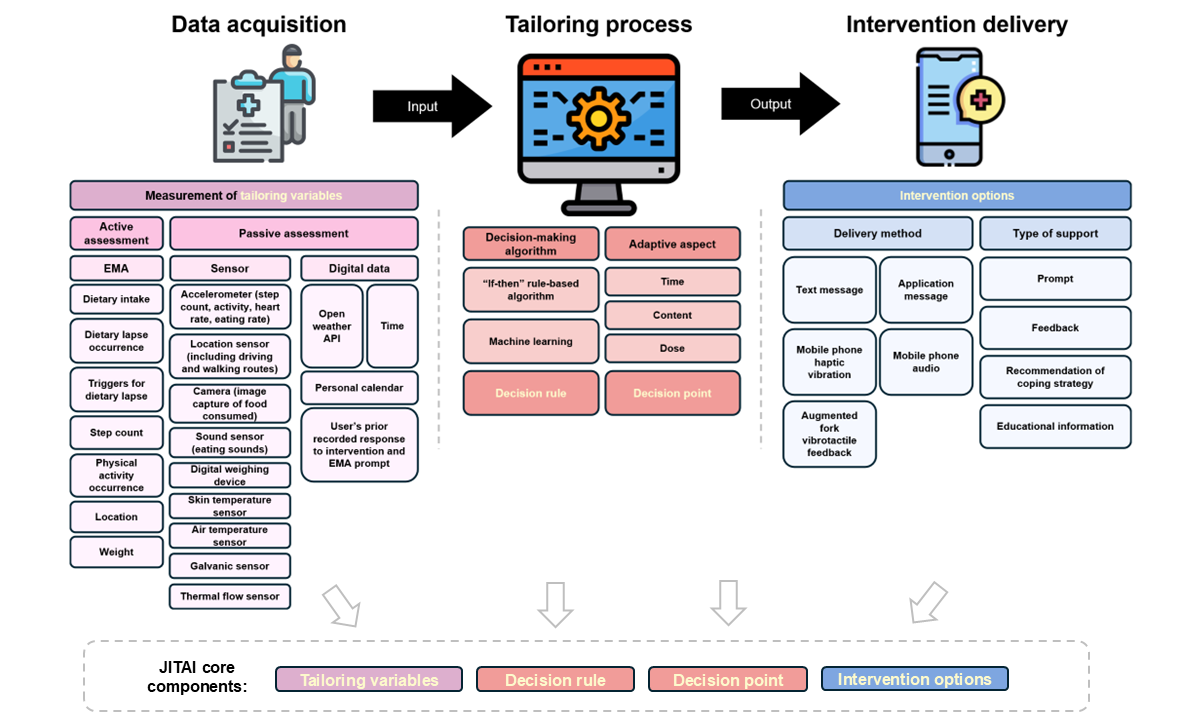
Overview of just-in-time adaptive intervention (JITAI) features. API: Application Programming Interface; EMA: Ecological Momentary Assessment; JITAI: Just-in-time Adaptive Intervention.

Across studies, JITAIs shared common design components, including data acquisition, algorithm-driven tailoring processes, and tailored intervention delivery. Figure 2 provides a visual summary of the JITAI features discussed in this review.

#### Data Acquisition

To measure tailoring variables, 14 studies used active assessments only [10,31-37,43,45,46,54], 12 studies used passive assessments only [25,41,42,44,49,52,53,55-59], and 9 studies used both (Tables 1 and 3) [3,26-30,39,40,47,48,50,51].

Active assessments included EMA methods such as user-initiated, time-based, and event-based EMA (n=8) [10,32-37,54]. Other studies incorporating active assessments also used self-reported weight measurements, and questionnaires focused on dietary intake, dietary lapses, physical activity, and location (n=15) [26-31,39,40,43,45-48,50,51]. Passive assessments primarily used accelerometers (n=19) [25-30,39-42,44,47-49,51-53,55,57] to measure physical activity (n=18) [25-30,39-42,44,47-49,51-53,55] and eating rate (n=1) [57]. Other sensors used were location sensors (n=4) [44,49,50,52], galvanic sensors (n=2) [41,42], thermal flow sensors (n=2) [41,42], skin temperature sensors (n=2) [41,42], air temperature sensors (n=2) [41,42], image capturing monitors (n=2) [53,58], sound sensors (n=1) [59], and digital weighing devices (n=2) [52,53], Time (n=10) [10,33-37,44-46,49], weather (n=2) [44,49], and personal calendars (n=2) [49,52] were also passively sampled to contextualize user behavior and enhance intervention relevance (Tables 1 and 3).

#### Tailoring Process

The JITAI systems used either basic “if-then” rule-based algorithms (n=24) [25-31,39-48,50,51,53,55-58] or machine learning algorithms (n=11; Tables 1 and 3) [10,32-37,49,52,54,59]. Among the 5 interventions using machine learning, Step Up Life [49] and ThinkSlim [54] used decision tree–based models, DietAlert [10,32-37] used ensemble methods that combined multiple models to enhance predictive accuracy, Sweetch [52] used an adaptive reinforcement learning approach to optimize message compliance for individual users in specific contexts, and ADM-IPA [59] implemented a deep belief network to classify dietary intake. These systems leveraged machine learning to recognize activity patterns, user routines, and contextual information, thereby enhancing personalization and improving predictive accuracy, allowing for adaptation to individual needs in real time. Two interventions reported model performance: DietAlert demonstrated accuracies ranging from 0.67 to 0.80, with specificity between 0.68 and 0.84 and sensitivity between 0.45 and 0.77 [32-35], while ADM-IPA reported a best accuracy of 0.74 [59] (Table 2). In contrast, the remaining 14 interventions [25-31,39-48,50,51,53,55-58] used “if-then” rule-based algorithms, in which predefined conditions triggered specific feedback or interventions (Table 2). These algorithms relied strictly on fixed rules and did not consider user behavior.

#### Intervention Delivery

Intervention delivery methods varied across the included studies. Application messages were most common (n=24) [10,25-30,32-37,39-42,44,47,48,50-52,54] followed by text messages (n=7) [31,43,45,46,55,57,58], audio feedback with or without an earpiece (n=3) [53,57,59], mobile phone haptic vibrations (n=1) [49], and vibrotactile feedback via an augmented fork (n=1) [56]. Smartphones were the primary device in all studies except one [56], with 16 out of 35 studies (46%) also using at least one wearable device, such as smartwatches, in addition to the primary device (Tables 1 and 3) [25-30,39-42,47,48,53,55,57,59].

The included JITAIs offered a range of types of support, including prompts, feedback messages, recommendations for coping strategies, and educational information. Most studies used a combination of prompts and feedback (n=17) [25-30,39,40,44,47-49,53,54,57-59]. Four studies used prompts exclusively [41,42,50,51], 2 studies relied solely on feedback [52,56], and 7 studies integrated prompts with recommendations for coping strategies [10,32-37]. Finally, 5 studies combined prompts, feedback, and educational information (Tables 1 and 3) [31,43,45,46,55]. Prompts were used in various ways, including reminders for physical activity or self-monitoring of weight or exercise [25,27,41,55], and served as alerts to notify users when their dietary behavior was approaching undesirable thresholds [43,50,54,56]. Feedback messages were designed to inform users about their achievements and areas needing improvement. For instance, messages might offer positive reinforcement for successfully limiting calorie intake while encouraging further reduction in sugar consumption [27]. Feedback could also include actionable suggestions tailored to the user in a real-time context, such as recommending physical activities based on location and time of day [44,52]. Recommendations for coping strategies included techniques such as cognitive restructuring and problem-solving [33,34]. Educational information was presented as factual content to users [31,43].

### User-Related Outcomes

#### User Experience

Retention rates were high across studies, ranging from 74% [29] to 100% [32,43,51]. Although 100% retention was most frequently reported in studies that provided participant compensation [32,51], one noncompensated study also achieved 100% retention [43]. Among noncompensated studies, retention ranged from 74% [29] to 100% ([43] Table 2). Definitions of compliance varied across studies but were most commonly based on whether participants viewed, opened, or responded to prompts or feedback. For example, the DietAlert studies defined compliance as EMA survey responses, which ranged from 62.9% to 94.6% [32-37]. In contrast, the SMARTER trial measured compliance using the proportion of feedback messages opened, which ranged from 42.2% to 65.3% [26,28,29,39]. Across all interventions, the reported compliance rate ranged from 15.1% to 94.6% (Table 2) [26,28,29,31-37,39,42,45,47,51,54]. Participants rated interventions as effective, useful, and motivating, with high satisfaction scores ranging from 84.6% to 90% and a strong willingness to recommend their use (Table 2) [31,33,34,41,44,45,49]. Five studies found that greater user engagement was associated with better weight loss outcomes [26,30,31,34,40]. Twenty-three studies incorporated elements to increase user engagement [10,25-31,33-37,39-42,45,46,49,50,52,55], such as creating diverse intervention options and addressing user availability. Preliminary feedback indicated that users preferred interventions with lower demands (eg, prompting a 3-minute break more frequently vs a 12-minute break less frequently [41,42]), milder feedback [40,48], and greater context-sensitivity [40] (Table 2).

#### Intervention Outcomes

Significant weight loss was reported across multiple interventions [26,28-31,33-37,39,40,43,45-47,52], ranging from 0.5% [36] to 3.7% [37]. Reductions in BMI, waist circumference, and blood pressure were also reported [28,29,52,56]. Improvements in physical activity were consistently observed across studies, including greater adherence to physical activity [39,52], decreased sedentary time [41], and increases in step count or walking minutes [25,31,42,55]. Dietary behaviors also improved, with decreases in dietary lapses [33,37], increased adherence to calorie goals [26], improvements in overall diet quality, such as higher fruit and vegetable intake [30,46], and slower eating rates [56] (Table 2).

## Discussion

### Principal Findings

This review synthesizes the limited yet diverse literature on JITAIs for weight management, clarifying their conceptual framework and highlighting dynamic approaches for future JITAI development. Key elements examined included behavioral theory adoption, distal and proximal outcomes, data acquisition, tailoring processes, intervention delivery methods, user experiences, and intervention outcomes. Based on these findings, we emphasize the significant potential of JITAIs for weight loss and aim to inform future researchers about the conceptual underpinnings, features, and effectiveness of implementing JITAIs for weight management.

### JITAI Conceptual Design and Outcomes

#### Behavioral Theories

Consistent with previous JITAI reviews, this review identified a lack of behavioral theories incorporated in weight management JITAIs, potentially stemming from application developers’ limited familiarity with health behavioral theories [8,14]. Multidisciplinary collaborations between engineers, behavioral scientists, and clinicians could bridge this gap [61]. In the absence of dynamic theories, the lack of even static ones is a major shortcoming, as they are essential for comprehending the intricacies of real-world behavior and the dynamics of behavioral change [62]. Theory plays a critical role in the design, implementation, and evaluation of behavior change interventions [63], and DBCIs that integrate theories have shown better outcomes than those that do not [8,62,64]. Theories are valuable in informing the selection of tailoring variables, decision rules, decision points, and intervention options [6,65].

#### Distal and Proximal Outcomes

Weight loss was the distal outcome for all studies, given that it was the inclusion criterion. The most common health behavior proximal outcome was dietary behavior. This is likely because dietary behavioral change has been found to have a more significant and consistent effect on weight loss outcomes than physical activity change, although the combination of both is more effective than either alone [66-68].

Other proximal outcomes included physical activity and self-weighing, which reflect the pathways through which weight management JITAIs can exert their effects. Notably, studies that used JITAI approaches to target both physical activity and dietary lapses simultaneously yielded more conclusive results regarding their effectiveness in promoting weight management behaviors [69]. Moreover, diet-focused JITAIs appear to be more successful when paired with other strategies, such as food logging and self-weighing [34]. Collectively, these findings highlight that while diet remains a primary factor in weight loss, a more integrated approach that incorporates physical activity, self-monitoring behaviors, and supportive strategies may maximize the effectiveness and sustainability of JITAIs.

### JITAI Features

#### Data Acquisition

Physical activity and eating rate were mostly measured with passive assessments, but for dietary structure assessment, most studies relied on EMAs. This suggests that passive food intake monitoring technology may not be as accessible as those for physical activity or eating rate [70]. However, a limitation of relying on EMAs over passive assessments is that they may increase cognitive burden and reduce engagement, especially if presented too frequently, as users grow weary of responding [6,10,12]. Possible alternatives for dietary structure assessment with lower user burden include the employment of image processing technology in active assessment, where users simply need to upload photos of their meals, or passive assessment with automatic image recognition through continuous video monitoring [58,70].

#### Tailoring Process

Most studies opted for rule-based algorithms as opposed to machine learning. Machine learning can optimize the strengths of JITAIs, enhancing predictive functions and increasing the accuracy of tailoring processes. In addition, the increased information relevance can increase perceived usefulness and user engagement [71]. Machine learning can also support the cost-effective upscaling of JITAI deployment [52]. However, integrating machine learning can be resource-intensive due to computational demands (eg, handling missing data, optimizing parameters, and model testing) and may not always be necessary or effective [10]. For example, machine learning has been effective in predicting lapses at a group level, but its strength falters at an individual level [72,73]. Machine learning also loses its significance if a causal relationship between 2 variables has already been established in the literature [10].

Furthermore, the no free lunch theorem posits that no algorithm is universally optimal for all problems, meaning machine learning is not a 1-size-fits-all solution, but rather a collection of approaches that can be tailored or personalized at the individual level to improve upon prespecified algorithms [74]. Therefore, while machine learning has significant potential to enhance JITAI effectiveness, developers should only use it when practical and likely to improve user experience. Developers should prioritize designing theory-based JITAIs based on feasibility and user needs.

#### Intervention Delivery

The types of support identified in the included studies were prompts, feedback, recommendations for coping strategies, and educational information. There were differing user preferences regarding the intervention options, with some users preferring more concise instructions [58] and others preferring more detailed ones to guide them [40]. A major challenge in designing effective intervention options is the need to consider variables including boredom, habituation, cognitive overload, and contextual restraints [6].

Given the numerous potential intervention options and delivery methods, as well as the ambiguous efficacy of each component, researchers could consider using microrandomized trials. Unlike traditional randomized controlled trials, participants are not assigned to groups. Instead, each JITAI intervention, and how the participant interacts with it, would be considered a trial. Components of the intervention, such as content, timings, and frequencies, would be randomized based on the study. Data on proximal outcomes, or immediate reactions to the intervention, would be collected and analyzed using regression models at the decision-point level. Information gathered would then be linked to distal outcomes, analyzed at a participant level instead [75].

Microrandomized trials allow researchers to account for the element of time in their study, while also collecting a large number of data points both between and within participants. In addition, insights can be gained from qualitative research, and reinforcement learning could be used to learn user preferences [75].

### User-Related Outcomes

#### User Experience

Ultimately, one of the greatest challenges JITAIs face is optimizing user engagement [10], as evidenced by the highly variable compliance rates found in the included studies, which are attributed to the continuous requirement for lengthy user inputs. Intrinsically, the JITAI design integrates elements accounting for states of receptivity (eg, availability, whether the user is driving, or sleeping), which should increase user engagement [6]. However, many of the studies did not include, or included insufficient, elements to address this. This includes both studies that termed themselves JITAIs and those that did not. We were unable to evaluate sustained engagement due to a lack of evidence, similar to findings from another review on JITAIs for physical activity [14]. This is likely because JITAIs are still in development, and intervention durations were relatively short. In addition, previous literature indicated that JITAIs often have short lifespans [50].

One way to improve user experience and engagement is by focusing on promoting long-term weight loss by enhancing self-efficacy and providing coping strategies, rather than relying solely on short-term prompt effects [76,77]. Similarly, incorporating passive sensing, especially for dietary habits, could reduce user burden and has been shown to reduce attrition rates [78]. User engagement can also be further improved by developing user-centered design elements, such as interfaces or interventions that have been personalized based on the social context of the users [79]. By integrating such features, JITAIs are likely to be perceived as more useful, engaging, and ultimately more effective in sustaining behavior change.

#### Intervention Outcomes

Across interventions, JITAIs demonstrated significant impacts on distal and proximal outcomes, such as weight loss, reductions in BMI, waist circumference, and blood pressure, as well as improvements in physical activity and dietary behavior. These outcomes reflect the ability and effectiveness of JITAIs to deliver adaptive, personalized, and timely support that targets the behavioral and contextual factors most relevant to each individual. By leveraging personalized data, JITAIs can improve weight management strategies by reducing sedentary time [41,42,55], encouraging adherence to calorie goals [26], and providing prompts that mitigate dietary lapses [10,32-37]. The dynamic and individualized features of JITAIs further help to sustain engagement and adherence, which are crucial for long-term behavior change [34,36,39,42,51]. Hence, these findings highlight the role of JITAIs in translating behavioral theory into practice, producing measurable improvements in lifestyle habits and health outcomes.

### Strengths and Weaknesses

Building on these findings, the studies included demonstrated several strengths. Many interventions leveraged active data from wearables and mobile devices to enable real-time, adaptive, and context-sensitive delivery of feedback. Over one quarter of the studies included incorporated both passive and active data [26-30,39,40,47,48,50,51], combining the advantages of passive sensing, such as capturing contextual or emotional states not detected by devices, with the strengths of active input, thereby reducing notification fatigue and increasing user engagement [44,50,52]. Personalization was also another key strength, with tailoring variables such as activity levels, location, weather, and personal calendars enhancing relevance and user engagement. Nearly half of the interventions [25-37,39-46,49,51,52,59] were grounded in behavioral theories, strengthening their conceptual rigor and enhancing the precision of the intervention to meet user needs and contexts. In addition, several studies used machine learning to analyze complex data streams and optimize decision rules [10,32-37,49,52,54,59], elucidating the growing role of artificial intelligence in enhancing intervention adaptivity. Collectively, these strengths highlight the promise of JITAIs to bridge theory and practice while providing scalable and personalized digital health solutions.

However, a few weaknesses remain. Most studies did not explicitly identify their designs as JITAIs, resulting in heterogeneous information types, with many lacking detailed descriptions of design components such as outcomes, decision rules, and decision points. This aligns with previous reports on the lack of JITAI terminology [8] and incomplete reporting in JITAI studies [16]. Given that JITAI is a relatively novel term, this was anticipated. However, the absence of comprehensive information hindered the in-depth evaluation of core components.

Furthermore, inconsistent terminology, incomplete reporting, a lack of empirical evidence, and the absence of established treatment protocols have impeded JITAI development [14]. Existing behavioral theories are static and may not adequately support JITAI advancement [6,8,60]. Although detailed frameworks have been published to guide JITAI design [7,80], their adoption appears to be limited due to complexity. A recently developed JITAI reporting checklist aims to provide more streamlined guidance [15], but its usefulness remains to be fully assessed.

Moreover, this review identified only one qualitative study [40], as most research focused primarily on development rather than evaluation. The limited use of qualitative methods limits insights into user perspectives and sentiment, which are critical for informing JITAI design and optimizing the effectiveness of DBCIs in enhancing health outcomes [81].

### Limitations

This review was limited to English-language studies, potentially excluding relevant research, particularly from technologically advanced regions such as China, thus affecting comprehensiveness and generalizability. The inclusion of studies not explicitly labeled as JITAIs introduced the possibility of error, despite adherence to established definitions of JITAIs. Due to resource constraints, a second reviewer was not involved in the screening process, increasing the risk of bias. Most included studies were developmental in nature, preventing assessment of weight loss outcomes. Finally, the review lacks software engineering perspectives on JITAIs, an area outside the authors’ expertise. Nonetheless, the review provides valuable insights into the potential of JITAIs as a public health tool for weight management.

### Implications

This review offers guidance for developers creating weight management JITAIs, encouraging the usage of terminology and design to support JITAI development and advance research. Future research should explore the optimization of JITAI components, such as through microrandomized trials, integration of behavioral theories, and machine learning. The review findings also reinforce the importance of user engagement; researchers are urged to optimize user engagement by understanding user needs, perceived usefulness, and states of receptivity. Although we conducted a preliminary mapping of intervention outcomes, sample sizes in most studies were small and highly variable (n=8502). Future research should therefore use larger and more consistent samples to provide more robust evidence and better evaluate the long-term effectiveness of JITAIs in weight management.

Nonetheless, weight management JITAIs have the potential to become a scalable and cost-effective approach to managing excessive body weight [82]. They can assist health care providers in supporting patients and extend care to individuals with limited access to traditional health care services, both locally and globally.

### Conclusion

This review highlights the role of JITAIs for weight loss and outlines key features for future developers, including behavioral theories, distal and proximal outcomes, data acquisition, tailoring processes, intervention delivery methods, user experience, and intervention outcomes. However, the field is still in its early stages, with inconsistent reporting on key design components and small sample sizes for rigorous evaluation of weight loss outcomes. Key challenges include optimizing user engagement, integrating behavioral theories, and achieving long-term effectiveness. As the field matures, JITAIs could become scalable and cost-effective tools for supporting diverse populations in achieving sustainable weight loss.

## Appendix

Multimedia Appendix 1 PRISMA-ScR (Preferred Reporting Items for Systematic reviews and Meta-Analyses extension for Scoping Reviews) checklist.

Multimedia Appendix 2 Comprehensive search strategy, eligibility criteria, study characteristics, and JITAI conceptual design and features.

## Abbreviations

ADM-IPA: Automated Diet Monitoring Intelligent Personal Assistant
DBCI: digital behavior change intervention
EMA: ecological momentary assessment
JITAI: just-in-time adaptive intervention
PRISMA: Preferred Reporting Items for Systematic Reviews and Meta-Analyses
PRISMA-ScR: Preferred Reporting Items for Systematic Reviews and Meta-Analyses extension for Scoping Reviews
PROSPERO: International Prospective Register of Systematic Reviews
WHO: World Health Organization

## Data Availability

Data sharing is not applicable to this article as no data sets were generated or analyzed during this study.
