# Supplementary material for: Just-In-Time Adaptive Interventions for Weight Management Among Adults With Excess Body Weight: Scoping Review
Source: J Med Internet Res. 2025 Dec 25;27:e76625. doi: 10.2196/76625 (PMC12784143; doi:10.2196/76625)
Supplement: Multimedia Appendix 2 [file jmir_v27i1e76625_app2.docx]

**Table S2.** Comprehensive search strategy.

| Database | Index and keyword terms | Results |
| --- | --- | --- |
| PubMed | #1: "just-in-time"[Title/Abstract] OR "JITAI*"[Title/Abstract] OR "ecological momentary intervention*"[Title/Abstract] OR "context-aware*"[Title/Abstract] OR "adaptive intervention"[Title/Abstract:~4] OR "adaptive interventions"[Title/Abstract:~4] OR "digital behavior change"[Title/Abstract] OR "digital behaviour change"[Title/Abstract] OR "DBCI*"[Title/Abstract] OR "dynamic tailoring"[Title/Abstract:~4] OR "dynamically tailored"[Title/Abstract:~4] OR "real-time tailoring"[Title/Abstract:~4] OR "real-time therap*"[Title/Abstract] OR "tailored feedback"[Title/Abstract:~4] OR "adaptive feedback"[Title/Abstract:~4] OR "real-time feedback"[Title/Abstract:~4] OR "microrandomi*"[Title/Abstract] OR "micro-randomi*"[Title/Abstract] | 10,191 |
|  | "Overweight"[Mesh] OR "Weight Loss"[Mesh] OR "Weight Reduction Programs"[Mesh] OR "Overweight"[Title/Abstract] OR "weight loss"[Title/Abstract] OR "weight reduction"[Title/Abstract] OR "weight management"[Title/Abstract] OR "body weight"[Title/Abstract] OR "obes*"[Title/Abstract] OR "overweight"[Title/Abstract] OR "BMI"[Title/Abstract] OR "body mass"[Title/Abstract] | 1,008,264 |
|  | #1 AND #2 | 381 |
| EMBASE | #1: 'obesity'/exp OR 'body weight loss'/exp OR 'body weight management'/exp OR 'body weight':ab,ti OR 'weight management':ab,ti OR 'weight loss':ab,ti OR 'weight reduction':ab,ti OR 'overweight':ab,ti OR 'obes*':ab,ti OR 'bmi':ab,ti OR 'body mass':ab,ti | 1,732,949 |
|  | #2: 'just-in-time':ab,ti OR 'jitai$':ab,ti OR 'ecological momentary intervention$':ab,ti OR 'context-aware*':ab,ti OR ((adaptive NEAR/5 intervention$):ab,ti) OR 'digital behavio$r change':ab,ti OR 'dbci$':ab,ti OR ((dynamic* NEAR/5 tailor*):ab,ti) OR (('real time' NEAR/4 tailoring):ab,ti) OR 'real-time therap*':ab,ti OR ((tailor* NEAR/5 feedback):ab,ti) OR ((adapt* NEAR/5 feedback):ab,ti) OR (('real time' NEAR/5 feedback):ab,ti) OR 'microrandomi*':ab,ti OR 'micro-randomi*':ab,ti | 14,648 |
|  | #1 AND #2 | 571 |
| Cochrane-Central  (Central Register of Controlled Trials) | #1: ("just-in-time" OR JITAI? OR (ecological NEXT momentary NEXT intervention?) OR context-aware* OR (adaptive NEAR/5 intervention?) OR (digital NEXT behavio?r NEXT change) OR dbci? OR (dynamic* NEAR/5 tailor*) OR (real-time NEAR/5 tailoring) OR (real-time NEXT therap*) OR (tailor* NEAR/5 feedback) OR (adapt* NEAR/5 feedback) OR (real-time NEAR/5 feedback) OR microrandomi* OR micro-randomi*):ti,ab,kw | 6246 |
|  | #2: ( "body weight" OR "weight management" OR "weight reduction" OR "weight loss" OR overweight OR obes* OR BMI OR "body mass" ):ti,ab,kw | 181424 |
|  | #3: MeSH descriptor: [Overweight] explode all trees | 25675 |
|  | #4: MeSH descriptor: [Weight Loss] explode all trees | 9228 |
|  | #5: MeSH descriptor: [Weight Reduction Programs] explode all trees | 1169 |
|  | #6: #2 OR #3 OR #4 OR #5 | 181115 |
|  | #7: #1 AND #6 | 789 |
| CINAHL | S1: TI ( "just-in-time" OR JITAI# OR "ecological momentary intervention*" OR context-aware* OR (adaptive N4 intervention*) OR "digital behavio*r change" OR DBCI# OR (dynamic* N4 tailor*) OR (real-time N4 tailoring) OR "real-time therap*" OR (tailor* N4 feedback) OR (adapt* N4 feedback) OR (real-time N4 feedback) OR microrandomi* OR micro-randomi* ) OR AB ( "just-in-time" OR JITAI# OR "ecological momentary intervention*" OR context-aware* OR (adaptive N4 intervention*) OR "digital behavio*r change" OR DBCI# OR (dynamic* N4 tailor*) OR (real-time N4 tailoring) OR "real-time therap*" OR (tailor* N4 feedback) OR (adapt* N4 feedback) OR (real-time N4 feedback) OR microrandomi* OR micro-randomi* ) | 3,353 |
|  | S2: TI ( "body weight" OR "weight management" OR "weight reduction" OR "weight loss" OR overweight OR obes* OR BMI OR "body mass" ) OR AB ( "body weight" OR "weight management" OR "weight reduction" OR "weight loss" OR overweight OR obes* OR BMI OR "body mass" ) | 259,318 |
|  | S3: (MH "Obesity+") OR (MH "Weight Loss+") OR (MM "Weight Reduction Programs") | 136,023 |
|  | S4: S2 OR S3 | 295,271 |
|  | S5: S1 AND S4 | 167 |
| PsycINFO | #1: ("just-in-time" or JITAI? or "ecological momentary intervention?" or context-aware* or (adaptive adj5 intervention?) or "digital behavio?r change" or dbci? or (dynamic* adj5 tailor*) or (real-time adj5 tailoring) or "real-time therap*" or (tailor* adj5 feedback) or (adapt* adj5 feedback) or (real-time adj5 feedback) or microrandomi* or micro-randomi*).ab,ti. | 4450 |
|  | #2: exp Overweight/ or exp Weight Loss/ | 34615 |
|  | #3: ("body weight" or "weight management" or "weight reduction" or "weight loss" or overweight or obes* or BMI or "body mass").ab,ti. | 92272 |
|  | #4: #2 OR #3 | 93445 |
|  | #5: #1 AND #4 | 147 |
| Scopus | ( TITLE-ABS-KEY ( "just-in-time" OR jitai* OR "ecological momentary intervention*" OR context-aware* OR ( adaptive W/4 intervention* ) OR "digital behavio*r change" OR dbci* OR ( dynamic* W/4 tailor* ) OR ( real-time W/4 tailoring ) OR "real-time therap*" OR ( tailor* W/4 feedback ) OR ( adapt* W/4 feedback ) OR ( real-time W/4 feedback ) OR microrandomi* OR micro-randomi* ) AND TITLE-ABS-KEY ( "body weight" OR "weight management" OR "weight reduction" OR "weight loss" OR overweight OR obes* OR bmi OR "body mass" ) ) | 650 |
| Web of Science | TI=("just-in-time" OR jitai* OR "ecological momentary intervention*" OR context-aware* OR ( adaptive NEAR/4 intervention* ) OR "digital behavio*r change" OR dbci* OR ( dynamic* NEAR/4 tailor* ) OR ( real-time NEAR/4 tailoring ) OR "real-time therap*" OR ( tailor* NEAR/4 feedback ) OR ( adapt* NEAR/4 feedback ) OR ( real-time NEAR/4 feedback) OR microrandomi* OR micro-randomi*) OR AB=("just-in-time" OR jitai* OR "ecological momentary intervention*" OR context-aware* OR ( adaptive NEAR/4 intervention* ) OR "digital behavio*r change" OR dbci* OR ( dynamic* NEAR/4 tailor* ) OR ( real-time NEAR/4 tailoring ) OR "real-time therap*" OR ( tailor* NEAR/4 feedback ) OR ( adapt* NEAR/4 feedback ) OR ( real-time NEAR/4 feedback) OR microrandomi* OR micro-randomi*) | 52,855 |
|  | TI=("body weight" or "weight management" or "weight reduction" or "weight loss" or overweight or obes* or BMI or "body mass") OR AB=("body weight" or "weight management" or "weight reduction" or "weight loss" or overweight or obes* or BMI or "body mass") | 1,119,905 |
|  | #1 AND #2 | 469 |
| ProQuest Dissertations & Theses | noft("just-in-time" OR jitai* OR "ecological momentary intervention*" OR context-aware* OR ( adaptive NEAR/4 intervention* ) OR "digital behavio*r change" OR dbci* OR ( dynamic* NEAR/4 tailor* ) OR ( real-time NEAR/4 tailoring ) OR "real-time therap*" OR ( tailor* NEAR/4 feedback ) OR ( adapt* NEAR/4 feedback ) OR ( real-time NEAR/4 feedback) OR microrandomi* OR micro-randomi*) AND noft("body weight" or "weight management" or "weight reduction" or "weight loss" or overweight or obes* or BMI or "body mass") | 60 |
| Science.gov | Title: (just-in-time OR jitai* OR "ecological momentary intervention" OR “ecological momentary interventions” OR context-aware* OR “adaptive intervention” OR “adaptive interventions” OR "digital behaviour change" OR “digital behavior change” OR dbci* OR “dynamic tailoring” OR “dynamically tailored” OR “real-time tailoring” OR "real-time therap" OR “tailored feedback” OR “adaptive feedback OR “real-time feedback” OR microrandomi* OR micro-randomi*) AND ("body weight" or "weight management" or "weight reduction" or "weight loss" or overweight or obes* or BMI or "body mass") | 61 |
| IEEE Xplore | #1: "All Metadata":"just-in-time" OR "All Metadata":jitai OR "All Metadata":JITAIs OR "All Metadata":"ecological momentary intervention" OR "All Metadata":"ecological momentary interventions" OR "All Metadata":context-aware* OR "All Metadata":adaptive NEAR/4 intervention* OR "All Metadata":"digital behavio*r change" OR "All Metadata":DBCI OR "All Metadata":DBCIs OR "All Metadata":dynamic* NEAR/4 tailor* OR "All Metadata":real-time NEAR/4 tailoring OR "All Metadata":tailor* NEAR/4 feedback OR "All Metadata":adapt* NEAR/4 feedback OR "All Metadata":real-time NEAR/4 feedback OR "All Metadata":microrandomi* OR "All Metadata":micro-randomi* | 33,974 |
|  | #2: "Mesh_Terms":overweight OR "Mesh_Terms":"obesity" OR "Mesh_Terms":"weight loss" OR "All Metadata":"body weight" OR "All Metadata":"weight management" OR "All Metadata":"weight reduction" OR "All Metadata":"weight loss" OR "All Metadata":overweight OR "All Metadata":obese OR "All Metadata":obesity OR "All Metadata":BMI OR "All Metadata":"body mass" | 11,233 |
|  | #3: #1 AND #2 | 184 |
| ClinicalTrials.gov | Overweight OR obes* OR BMI/Body Composition OR "Body Weight" OR "weight loss" OR "Weight Reduction" OR "Weight Management" \| Just-In-time Adaptive Intervention \(JITAI\) OR just-in-time OR Adaptive Intervention \(AI\) OR “Ecological Momentary Intervention” OR context-aware* OR "digital behavior change" OR "digital behaviour change" OR dbci* OR "dynamically tailored" OR "dynamic tailoring" OR "real-time tailoring" OR "real-time therapy" OR "tailored feedback" OR "adaptive feedback" OR "real-time feedback" OR microrandomi* OR micro-randomi* | 97 |
| ICTRP | Condition: (Overweight OR obes* OR BMI OR “body mass” OR "Body Weight" OR "weight loss" OR "Weight Reduction" OR "Weight Management") \| Intervention: (just-in-time OR “Adaptive Intervention” OR “adaptive interventions” OR JITAI* OR “Ecological Momentary Intervention” OR “ecological momentary interventions” OR context-aware* OR "digital behavior change" OR "digital behaviour change" OR dbci* OR "dynamically tailored" OR "dynamic tailoring" OR "real-time tailoring" OR "real-time therapy" OR "tailored feedback" OR "adaptive feedback" OR "real-time feedback" OR microrandomi* OR micro-randomi*) | 31 |
| ISRCTN | (Overweight OR obes* OR BMI OR "body mass" OR "Body Weight" OR "weight loss" OR "Weight Reduction" OR "Weight Management") AND (just-in-time OR "Adaptive Intervention" OR "adaptive interventions" OR JITAI* OR "Ecological Momentary Intervention" OR "ecological momentary interventions" OR context-aware* OR "digital behavior change" OR "digital behaviour change" OR dbci* OR "dynamically tailored" OR "dynamic tailoring" OR "real-time tailoring" OR "real-time therapy" OR "tailored feedback" OR "adaptive feedback" OR "real-time feedback" OR microrandomi* OR micro-randomi*) | 18 |

**Table S3.** Eligibility criteria.

| Criteria | Inclusion criteria | Exclusion criteria |
| --- | --- | --- |
| Population | Overweight (BMI ≥ 25 kg/m^2^)  Adults (Age ≥ 18)  Conceptual/feasibility/pilot studies, which do not limit BMI or age but explicitly address weight management, are also included | Not overweight (BMI <25kg/m^2^)  Adolescents or children (<18 years old) |
| Concept | - Just-in-time adaptive interventions must minimally have the following 2 components:   1. Adaptation of intervention delivery: (a) Adapts the provision of support (can be content/dose/timing), (b) over time to an individual’s changing status and contexts to deliver support, (c) At the moment and in the context that the person needs it most and is most likely to be receptive   2. Real-time monitoring: Monitor the dynamics of an individual’s internal state and context in real time. The goal is to identify the states of vulnerability/opportunity - Digital Behavior Change Intervention - Minimally partially automated - Support is tailored to be provided when the user is in a state of vulnerability/opportunity - Intended to motivate behavioral change | - Systems that do not provide prompts and instead require the participant to interact with the system at their own will and decision - Studies that focus on the real-time monitoring features, but barely describe the intervention delivery, if at all (suggesting that it is not truly a JITAI) - Studies that mention that they are related to another paper on JITAI, but do not discuss the JITAI design or its outcomes - Purely technical papers which do not include any rationales, concepts, or applicability of the application |
| Context | Weight management | - Mental health conditions - Substance abuse - Alcohol abuse - Smoking cessation - Malnutrition - Eating disorders - Physical rehabilitation |
| Type of design | All study designs except for reviews and frameworks:   - Feasibility study - Developmental papers - Conceptual papers - Pilot study - Experimental - Non-experimental - Case study - Secondary data analysis | Reviews  Frameworks |
| Year of publication | Until 2024 |  |
| Publication type | Journal articles  Theses  Conference abstracts  Conference proceedings |  |
| Language | English |  |

**Table S4.** Study characteristics (N=35).

| Author, Year | Country; Publication Type | Study Design; n | Sample Characteristics | Duration of intervention: baseline/training period | Aim | App used | Compliance | Key Outcomes |
| --- | --- | --- | --- | --- | --- | --- | --- | --- |
| Goldstein et al., 2016 [38] | USA; Thesis Dissertation | Pilot trial; n=12 | 18-65 years of age BMI 27-45 kg/m^2^  11 (91.7%) Female 6 (50%) Caucasian | 4 weeks (test model performance); 2 weeks (training set and data collection) | Build and evaluate ML model for lapse prediction | DietAlert + WW | 94.6% EMA survey response; 100% retention (with compensation) | Accuracy of model 0.67–0.72; Specificity of model 0.68–0.72; Sensitivity of model 0.45–0.70; more data ↑ model outcomes, especially sensitivity |
| Goldstein et al., 2017 [10] | USA; Journal Article | Developmental; NA | NA | 4 weeks (test model performance); 2 weeks (training set and data collection) | Applying the JITAI framework to OnTrack | DietAlert + WW | NA | NA |
| Forman et al., 2019a [37] | USA; Journal Article | Pilot; n=43 | 18-65 years of age BMI 25-50 kg/m^2^  37 (86%) Female 32 (74.4%) Caucasian | 8 weeks; 2 weeks | Feasibility, acceptability & effectiveness of OnTrack | OnTrack (formally DietAlert) + WW | 85.1% EMA survey response; 97.7% retention | Negative predictive value of model 80%; 70.15% alerts opened; 3.13% weight loss; app was easy to use, had minimal issues, moderately useful and enjoyable^1^; unplanned lapses ↓ over time |
| Forman et al., 2019b [36] | USA; Journal Article | RCT; n=181 | 18-70 years of age BMI 25-50 kg/m^2^  154 (85.1%) Female 133 (73.5%) Caucasian | 10 weeks; 2 weeks | Additive effect of OnTrack + WW vs WW alone and assessing lapse frequency, algorithm accuracy, and engagement | OnTrack (formally DietAlert) + WW | 62.9% EMA survey response; 88.4% retention | Specificity of model 83.8%; Sensitivity of model 69.2%; 46.9% alerts opened; 2.1% weight loss when moderated by diet type^; high satisfaction of app reported^1^; 72.8% risk alerts received as helpful/accurate; lapses ↓ over time |
| Goldstein et al., 2020 (Primary) [41] | USA; Journal Article | Randomized trial; n=121 | 18-70 years of age BMI 25-50 kg/m^2^  102 (84.3%) Female 89 (73.6%) Caucasian | 10 weeks; 2 weeks | Effect of EMA survey length (8 qns vs 17 qns) on algorithm performance, app utilization and behavioral outcomes | OnTrack (formally DietAlert) + WW | 65.4% (8 questions), 60.5% (17 questions) EMA survey response; 84.3% retention | Accuracy of model 79.7% (8 qns) vs 79.9% (17 qns); Specificity of model 84.4% (8 qns) vs 81.7% (17 qns); Sensitivity of model 71.6% (8 qns) vs 77.7% (17 qns); 46.9% alerts opened; 3.4% weight loss; 72.84% risk alerts received as helpful/accurate |
| → Goldstein et al., 2021a (Secondary) [39] | USA; Journal Article | Secondary analysis of randomized trial; n=121 | 18-70 years of age BMI 25-50 kg/m^2^  102 (84.3%) Female 89 (73.6%) Caucasian | 10 weeks; 2 weeks | Association between lapse frequency & weight loss, evaluating engagement with OnTrack | OnTrack (formally DietAlert) + WW | 63.1% EMA survey response | 0.49% weight loss; 50.3% accessed intervention library/week; lapse frequency not statistically significantly associated with percentage weight loss |
| → Goldstein et al., 2021b (Secondary) [40] | USA; Journal Article | Secondary analysis of randomized trial; n=121 | 18-70 years of age BMI 25-50 kg/m^2^  102 (84.3%) Female 89 (73.6%) Caucasian | 10 weeks; 2 weeks | Assess impact of planned/unplanned lapses and lapse types on weight loss; characterize lapse types by individual/contextual factors | OnTrack (formally DietAlert) + WW | 62.9% EMA survey response | Lapses occurrence: multiple 28.5% (Lasso accuracy 69.8%), planned 22.9% (67.2%), off-plan 16.4% (67.9%), larger portion 12.5% (67.7%), unknown points 10.8% (70.6%), unintended time 8.1% (69.0%); ↓unplanned lapses (larger portion, unintended time, multiple) ↑ weight loss; 3.7% weight loss observation |
| Burke et al., 2017 [29] | USA; Journal Article | Pilot randomized trial; n=39 | ≥18 years of age BMI ≥ 27 and ≤43 kg/m^2^  26 (67.2%) Female 33 (84.6%) Caucasian | 12 weeks | Feasibility of daily tailored feedback to dietary recordings and comparison of SM, SM+FB, and SM+FB+F2F | SMARTER + LoseIt! | SM 53.5%, SM+FB 55.9%, SM+FB+F2F 65.3% adherent to self-monitoring; 74% retention (with compensation) | All groups lost weight & ↓SBP; no between-group differences for weight loss, SBP, DBP and self-efficacy; DBP ↓ in SM & SM+FB+F2F; ↑ self-efficacy only in SM |
| Burke et al., 2020 [27] | USA; Journal Article | Developmental; NA | NA | 12 months; 5 days baseline prior to start of intervention with just the Fitbit SM (without SMARTER app with feedback) | Describe SMARTER study design, methods & framework | SMARTER + Fitbit | NA | NA |
| Burke et al., 2022 [28] | USA; Journal Article | RCT; n=502 | ≥18 years of age BMI ≥ 27 and ≤43 kg/m^2^  399 (79.5%) Female 423 (84.3%) Caucasian | 12 months; 5 days baseline prior to start of intervention with just the Fitbit SM (without SMARTER app with feedback) | Efficacy of SM+FB vs SM on weight loss at 6 months | SMARTER + Fitbit + 1-to-1 90min dietary counselling | 54.8% feedback messages opened; 86% retention | Both groups lost weight, ↓BMI and WC; no between-group differences. |
| Burke et al., 2022 (Primary) [26] | USA; Journal Article | RCT; n=502 | ≥18 years of age BMI ≥ 27 and ≤43 kg/m^2^  399 (79.5%) Female 423 (84.3%) Caucasian | 12 months; 5 days baseline prior to start of intervention with just the Fitbit SM (without SMARTER app with feedback) | Efficacy of SM+FB vs SM on weight loss at 12 months | SMARTER + Fitbit + 1-to-1 90min dietary counselling | 42.2% feedback messages opened; 78.5% retention | Both groups lost significant weight (–2.16kg); no between-group weight loss difference; % days adherent to calorie goal higher & declined slower in SM+FB; ↑feedback messages opening → ↑adherence to calorie goal and weight loss |
| → Cheng et al., 2023 (Secondary) [30] | USA; Journal Article | Secondary analysis of RCT; n=502 | ≥18 years of age BMI ≥ 27 and ≤43 kg/m^2^  399 (79.5%) Female 423 (84.3%) Caucasian | 12 months; 5 days baseline prior to start of intervention with just the Fitbit SM (without SMARTER app with feedback) | Assess the association between diet quality & weight loss in SM+FB vs SM | SMARTER + Fitbit + 1-to-1 90min dietary counselling | – | Minimal diet quality improvement overall; weight loss ≥5% linked to higher HEI-2015 scores at 6 mo but not sustained at 12 mo |
| → Bizhanova et al., 2022 (Secondary) [32] | USA; Journal Article | Secondary analysis of RCT; n=502 | ≥18 years of age BMI ≥ 27 and ≤43 kg/m^2^  399 (79.5%) Female 423 (84.3%) Caucasian | 12 months; 5 days baseline prior to start of intervention with just the Fitbit SM (without SMARTER app with feedback) | Identifying predictors of PA adherence | SMARTER + Fitbit + 1-to-1 90min dietary counselling | 66.5% met PA goal at wk1; median adherence higher in SM+FB (165.2%) vs SM (106.3%) at 12 mo but no within group differences; adherence non-linear and non-significant in both groups | Greater PA adherence linked to male sex, more feedback message engagement, higher baseline self-efficacy, week-1 adherence, greater weight loss at wk4 and 12mo, and fewer mental health issues; ML models (random forest regression, regression tree model, LASSO model) identified week-1 PA attainment as the strongest predictor |
| Kariuki et al., 2024 [33] | USA; Journal Article | Qualitative study; 6 focus groups; n=23 | ≥18 years of age BMI ≥ 27 and ≤43 kg/m^2^ | Not stated | Perceived utility and limitations of SMARTER in promoting PA and weight loss | NA | NA | Successful weight loss group: SMARTER helpful, organized, effective with consistent use; Unsuccessful weight loss group: weight gain or no weight loss experienced, feedback discouraging/harsh/out of context; Overall: both diet and PA are key in weight loss; most messages inaccessible due to 1-hr window, timing and personalization needed for such applications |
| Bond et al., 2014 (Primary) [34] | USA; Journal Article | Quasi study; n=35 | 21-70 years of age BMI ≥ 25 kg/m^2^ | 3 weeks (1 week per condition); 1 week | Test smartphone prompts for PA breaks on reduction of sedentary time | B-MOBILE | Not stated  85.7% retention (with compensation) | ↓ sedentary time, ↑ light & MVPA in all conditions; 3-min breaks gave largest effects (–5.9% sedentary time, +3.9% light PA); 90% found real-time feedback motivating and found smartphone prompts helpful in reducing sedentary time; 6-min PA breaks most preferred. |
| → Thomas et al., 2015 (Secondary) [35] | USA; Journal Article | Secondary analysis of quasi study; n=35 | 21-70 years of age BMI ≥ 25 kg/m^2^ | 3 weeks (1 week per condition); 1 week | Examine adherence of walking breaks from prompt, latency between prompts and beginning of walking breaks, and total walking minutes across 3-, 6-, 12-min PA prompts | B-MOBILE | Adherence: 3-min 89.4%, 6-min 86.7%, 12-min 77.0%. | Engagement was high across all conditions; 3-min prompts produced the most prompts/day (7.0), walking breaks/day (6.5), and shortest latency (23.0min); Walking minutes significantly different in 3- (37.2mins)and 6-min (38.7mins) condition vs 12-min (32.5mins); 3-min yielded similar total walking time as 6-min as participants often exceeded required duration of prompt |
| Westenenk et al., 2023 [25] | The Netherlands; Thesis Dissertation | Micro-randomized trial; n=13 | ≥ 18 years of age BMI ≥ 30kg/m^2^  10 (77%) Female | 14 days | To design a JITAI for PA, and to test how 0, 1, or 2 daily prompts and their timingaffect PA to identify the most effective delivery approach. | Ancora Health | 92.9% retention | Sending 1 prompt ↑ steps vs sending no prompt; evening prompts ↓ steps vs morning; 2 prompts/day ineffective. |
| Shapiro et al., 2012 [31] | USA; Journal Article | RCT; n=170 | 21-65 years of age BMI 25-39.9 kg/m^2^  110 (65%) Female 109 (64%) Caucasian | 12 months | Evaluate SMS/MMS + website vs control for weight loss | Text4Diet | ↑adherence to knowledge testing, ↓adherence to first and follow up weight and step query over time  76% retention (with compensation) | Modest weight loss, no group differences; higher SMS adherence → greater weight loss; ↑ steps linked to ↑ weight loss at 12 mo; moderately strong satisfaction with the program; ↑ satisfaction with pedometer component ↑ weight loss at 6 months; 85% would pay $4.99/mo. |
| Haggerty et al., 2016 [45] | USA; Journal Article | Randomized trial; n=20 | ≥18 years of age BMI > 30kg/m^2^ | 6 months | Assess feasibility and weight loss using telemedicine (weekly calls + WiFi scale) vs text messaging (Text4Diet) in women at risk for endometrial cancer, and examine pre–post changes in cancer-related biomarkers | Text4Diet | 100% retention | 90% lost weight; telemedicine > texting (-7.6% vs -4.1%); ↓ IL-2 post-intervention |
| Gupta & Sood, 2015 [42] | India; Conference Paper | RCT; n=33 | 24-30 years of age BMI ≥ 25kg/m^2^ | 4 weeks | Designing a context-aware app to encourage PA and testing the effectiveness and satisfaction of it | Let’s Exercise | Not stated | App rated effective/useful; 84.6% satisfied; 60% would continue using the app |
| Patrick et al., 2009 (Primary) [44] | USA; Journal Article | RCT; n=65 | 25-55 years of age BMI 25-39.9kg/m^2^  52 (80%) Female 49 (75%) Caucasian | 4 months | Develop and evaluate text message-based program vs usual care | mDIET | 2/3 messages responded by participants at the end of 4 months.  Retention 83.3% | Intervention lost –3.16% of weight vs –1.01% control; satisfaction high (92% would recommend) |
| →Norman et al., 2012 (Secondary) [43] | USA; Journal Article | Secondary analysis of RCT; n=65 | 25-55 years of age BMI 25-39.9kg/m^2^  52 (80%) Female 49 (75%) Caucasian | 4 months | Examining fruit and vegetable intake and eating behaviour as mediators of weight loss | mDIET | - | Intervention improved weight change, fruit and vegetable intake, and eating behaviour; fruit and vegetable intake and eating behaviour inversely associated with weight loss and were mediators of weight loss |
| Valle, Nezami & Tate, 2020 (Primary) [47] | USA; Journal Article | Micro-randomized trial; n=53 | 18-35 years of age BMI 25-40kg/m^2^  42 (79%) Female 33 (62%) Caucasian | 12 weeks | Characterize engagement with in-app messages in a weight management intervention | Nudge | 2/3 messages viewed by participants.  Retention 98.1% | Message viewing declined weekly (–0.15/day) and with weight gain (+1lb = –0.08) or longer lapses in weighing (–0.063/day). Viewing also fell with activity tracking lapses (–0.03/day). Likelihood of message viewing increased with more prior messages viewed (+0.07 per 1%), and more days meeting diet goals (+0.14/day). |
| →Hurley et al., 2024 (Secondary) [46] | USA; Journal Article | Secondary analysis of micro-randomized trial; n=53 | 18-35 years of age BMI 25-40kg/m^2^  42 (79%) Female 33 (62%) Caucasian | 12 weeks | Assess message viewing likelihood by unmet daily behavioural goals | Nudge | – | Each unmet goal ↓ odds of viewing messages by 34.8%. Odds of viewing also declined daily (OR = 0.977). Baseline depressive symptoms did not moderate these effects. |
| Rajanna et al., 2014 [48] | USA; Conference Paper | Developmental; user study n=4, formative evaluation n=2, summative evaluation n=2 | Age range: 20-35  4 (50%) Female | Not stated | To develop a smartphone application that promotes brief exercise after inactivity with PA reminders, evaluated through user studies. | Step Up Life | - | Around 80% of users from the 3 evaluation phases (user study, formative evaluation, summative evaluation) showed strong interest in the app. |
| Van Beurden et al., 2021 [49] | UK; Journal Article | Developmental; NA | NA | NA | Describe the systemic development of an app targeting impulsive processes for eating self-regulation to facilitate weight loss | ImpulsePal | NA | NA |
| Chen et al., 2024 [50] | USA; Conference Paper | \| Quasi-experimental (posttest only comparison group design); n=30 \| \| --- \| | 18-70 years of age  10 (33%) Female 5 (16%) Caucasian | 4 weeks | To investigate how the timing of the notifications (context- vs time-based) affects user engagement in an mHealth app | GatorTrack +  FatSecret | Completed 57.14% daily logs; 15.09% notification clicks; 18% notifications completed  100% retention (with compensation) | Context-based notifications led to higher click response time (12.33 vs 18.42 mins), click rate (19.05% vs 13.96%) and completion rates (21.77% vs 17.32%).  Average overall log rate greater in context-based condition (58.87% vs 55.54%) but not significant. |
| Everett et al., 2018 {Everett, 2018 #50 | USA, Journal Article | Quasi-experimental (one-group pretest-posttest); n=55 | BMI between 24 to 40kg/m^2^  33 (60%) Female 39 (71%) Caucasian | 3 months | Evaluate the feasibility, acceptability, safety, and effectiveness of the Sweetch app | Sweetch | 86% retention | ↑PA (2.8 metabolic equivalent task h/wk), ↓weight (–1.6kg, 2%), ↓BMI (–0.6kg/m2), ↓WC (–1.4cm), ↓A1c (–0.1%); high acceptability (78%) |
| Purpura et al., 2011 [52] | USA; Conference Paper | Randomized trial (conceptual); n=26 | Not stated | Not stated | Explore the Fit4Life persuasive system and ethical implications | Fit4Life (fictional) | NA | Focuses on ethical reflection, not efficacy |
| Spanakis et al., 2017 [53] | The Netherlands; Journal Article | Mixed-design (Study I developmental, Study II RCT); n=100 | Not stated | Study I 2 wks, Study II 8 wks | Present an algorithmic process that utilizes EMA methods for developing an ML approach to provide adaptive semi-individualized feedback to users regarding their eating behaviour | ThinkSlim | Study I: 80–81% assessments done; Study II: 70.5% assessments done, ~9.9h application usage | ML clustered 6 eater types; Study II showed the feasibility of adaptive feedback based on the 6 eater types |
| Finkelstein et al., 2015 [56] | USA; Conference paper | Pilot, randomized crossover study; n=30 | Adult women BMI > 30 kg/m^2^  30 (100%) Female 14 (47%) Caucasian | 8 weeks; 2 days | To assess whether a mobile app with real-time inactivity monitoring and tailored messaging (“message on” vs “message off”) can reduce sedentary time in overweight women | Fitbit One + Android smartphone app | 90% retention | Inactivity ↓during “message-on” periods (24.6% vs 30.4%).  Step count ↑ during “message-on” (6199 vs 5615 steps).  The majority of the participants expressed high acceptance and willingness to use the app in the future. |
| Hermsen et al., 2019 [54] | The Netherlands; Journal Article | RCT; n=141 | ≥18 years of age BMI ≥ 25kg/m^2^  69 (48.9%) Female | 5 weeks; 1 week | To examine the effects of 10sFork – a fork that gives vibrotactile feedback +/- retrospective visual feedback via an online dashboard on eating rate and body weight | 10sFork | 86.5% retention (with compensation)  In the group using the online dashboard, only 55.3% used. | Eating slowed (−1.8 bites/min), success ratio of >10s between bites ↑ 22.5%, BMI ↓ 0.5–0.8; dashboard added no benefit.  Vibrotactile feedback had a small to moderate effect on bite rate, a moderate to large effect on success ratio, and both remained significant for 8 weeks. |
| Mendi et al., 2013 [57] | Turkey; Conference Paper | Developmental; NA | BMI ≥ 25kg/m^2^ | NA | To develop a food intake monitoring system using real-time eating trends to manage weight loss | Android app + wrist-worn sensor | NA | NA |
| Moses et al., 2023 [58] | USA; Conference Paper | Mixed method (pilot quantitative study and qualitative study); n=29 | 18-65 years of age BMI 27-45 kg/m^2^  20 (69%) Female | Not stated | Discussing insights on the design and use of SMS messages to aid in changing eating behaviors for participants based on a user’s eating rate and energy intake. | SMS JITAI (energy intake, eating rate; numeric vs non-numeric) | Not stated | Participants preferred non-numeric messages (non-significant), but numeric messages were still positively received.  Statistical significance between message type and comprehension error rates. |
| Gao, 2021 [55] | USA; Thesis Dissertation | Quasi (one-group posttest); n=28 (lab testing), n=4 (in-the-field testing) | Not stated  8 (25%) Female | Not stated; At least 9 hours of video recordings per participant | Developing an automated diet monitoring intelligent personal assistant (IPA) system using exploratory study data from another IPA (Amazon Echo). | Automated Diet Monitoring IPA (ADM-IPA) | Not stated | ADM-IPA eating detection accuracy: 76.82% (in-the-field result), 94.57% (lab result) using Leave One Person Out analysis (in-the-field result), 87.22% (in-the-field result), 96.03% (lab result), using Leave One Sample Out analysis  >43% segments detected as eating activities, and the best accuracy obtained is 74.3%. |

DBP: diastolic blood pressure.

EMA: ecological momentary assessment.

FB: feedback.

F2F: face-to-face.

HEI-2015: Healthy Eating Index 2015.

JITAI: just-in-time adaptive intervention.

LASSO: least absolute shrinkage and selection operator.

ML: machine learning.

MVPA: moderate to vigorous physical activity.

NA: not applicable.

PA: physical activity.

RCT: randomized controlled trial.

SBP: systolic blood pressure.

SM: self-monitoring.

WC: waist circumference.

WW: Weight Watcher.

**Table S5.** JITAI conceptual design and features (N=35).

| Name of Intervention | Delivery | Intervention | Proximal Outcome | Theory | Algorithm | Intervention flow |
| --- | --- | --- | --- | --- | --- | --- |
| DietAlert / OnTrack [10, 36-41] | Active (event and time-based EMA prompts + user-initiated EMA) | Prompts + recommendations for coping strategies, via application messages  Event and time-based EMA 6×/day on lapse triggers; risk alerts with coping strategies; intervention library available on demand | Dietary lapses | Not stated | ML (logit boost, bagging, random subspace, random forest, Bayes net to form models); 2-week group data to train machine → individual adaptation in real time | EMA reply → algorithm predicts lapses (classification + variable selection model). If high risk: app delivers risk alert with top triggers + 3 coping strategies; user selects strategy for detailed intervention with interactive input. If no lapse predicted, no alert sent. |
| SMARTER  [26-30, 32, 33] | Active (FitBit self-reporting diet + weight)  Passive (FitBit PA) | Prompt and feedback via application messages  3 tailored messages/day on PA/diet; weight feedback every 6–8d; monthly message rotation | Diet, PA, weight SM | Kanfer’s Self-Regulation Theory, Social Cognitive Theory | Rule-based (self-report and passive data triggers tailored messages) | Participants log diet in Fitbit app, wear Fitbit tracker, and use wireless scale → data syncs to server where algorithm calculates progress, matches to a condition, and selects tailored message from library → messages delivered via SMARTER app. |
| B-MOBILE[34, 35] | Passive (smartphone + SenseWear Mini Armband) | Prompts only via application messages  Prompts for 3, 6, or 12-min PA breaks after 30–120 min sedentary behaviour | ↑ PA, ↓ sedentary time | Not stated | Rule-based (sedentary mins and PA occurrence triggers prompt) | Participants used a smartphone dashboard showing a fuel gauge of sedentary minutes and odometers for daily sedentary and activity time → sedentary limit reached → phone prompted a PA break with encouragement → participants could act, snooze, or dismiss → Completing a break triggered praise, a bright green ‘go’ light on the dashboard, and reset of the fuel gauge. |
| Ancora Health  [25] | Passive (smartphone or wearable) | Prompt and feedback via application messages  Step goal feedback messages; tailored to step goal achievement, phase of change (intender/actor) and time of day | Step count | Health Action Process Approach | Rule-based (set of 3 decision rules based on the 3 tailoring variables) | Push notifications give motivational or positive feedback based on step goal attainment 🡪 goal attainment (or no attainment) trigger intender vs actor messages (improving action vs habit formation)🡪 messages tailored by time of day (morning, afternoon, evening). |
| Text4Diet (modified version of mDIET)  [31, 45] | Active (self-reported weight + step count, knowledge-based questions) | Prompt, feedback and educational information via text messages  SMS/MMS 4×/day for 12 mo | Step count, weight self-monitoring | Social Cognitive Theory | Rule-based (user reply triggers tailored SMS) | 2000 unique SMS on tips, facts, motivation messages, knowledge questions, self-monitoring data, sent daily at random 🡪 users reported weight weekly and steps daily 🡪 personalized feedback via graphical MMS charts and daily pedometer goal, weekly encouragement provided. |
| Let’s Exercise  [42] | Passive (smartphone sensors: location, weather, time) | Prompt and feedback via application message  Positive/motivational messages + context-based PA recommendations (to encourage PA, round robin selection of messages) | PA | Not stated | Rule-based (from sensor trends) | Sensor data → rule aggregator (creates pattern) → pattern message database → alert triggered→ user response (choosing to respond now, later, too busy, won’t respond) → response stored to track trends |
| mDIET  [43, 44] | Active (self-reported weight, knowledge-based, eating behaviour questions) | Prompt, feedback and educational information via text messages  SMS/MMS (>3000 unique messages; half interactive) + weekly weight change graphs | Weight self-monitoring | Not stated | Rule-based (1500 decision rules created) | Message database → user-customized timing/frequency → daily diet/weight messages + tips/suggestions/encouragement + weekly weight & goal-setting |
| Nudge  [46, 47] | Active (weight, diet)  Passive (PA via activity tracker) | Prompt and feedback only via application messages  Daily prompts (max 1 per behaviour/day) for self-monitoring, encouragement, feedback or goal achievement | Weight self-monitoring, diet, PA | Not stated | Rule-based micro-randomization (4 decision points; 7 message types; 50/50 send) | App provided weekly lessons, tailored feedback, and progress pages 🡪 algorithm checks eligibility for 7 message types (weight, diet, activity) 🡪 randomly selects one 🡪 micro-randomized with 50% chance of sending 🡪 if sent, users prompted to view the message in Nudge. |
| Step Up Life [48] | Passive (smartphone accelerometer, age, location, time, weather, calendar) | Prompt and feedback via mobile phone haptic vibrations  Haptic vibration + exercise suggestion | PA | Fogg Behaviour Model | ML (decision tree) | App tailors exercise prompts by age, location, time of day, weather, and calendar 🡪 idle time thresholds adapt through the day based on time of day and amount of activity done 🡪 when inactivity exceeds the threshold, an alert with haptic vibration and exercise suggestion is sent 🡪 user can exercise (activity logged, threshold reset), snooze (prompt rescheduled), or cancel (counter updated, threshold reset) 🡪 interface gives positive feedback with rewards and animations. |
| ImpulsePal [49] | Active (emergency button – to be pressed when strong cravings experienced)  Passive (location) | Prompts only via application messages  Notifications (danger zones – at high risk locations, cravings/temptations experienced, inactivity of brain training game) | Resisting temptations and cravings | Not stated | Rule-based (sending messages based on location, inactivity or emergency button pressed) | App has 5 components. (1) Brain training - 5-min Go/No-go inhibition game, 3×/week for 4 weeks. (2) My Plan – user-selected or self-created if-then plans. (3) Urge surfing – mindfulness strategies for cravings. (4) Danger zones – notifications when entering high-risk locations. (5) Emergency button – press during cravings; provides visuospatial distraction, follow-up questions, and congratulatory feedback. A stats page tracks button use, success rate, and links to other strategies. |
| GatorTrack + FitBit  [50] | Active (diet, PA, weight logs, self-rated measures of weight related variables)  Passive (PA transitions) | Prompts only via application messages  Daily/weekly notifications on self-monitoring data and when PA transition is detected | Diet, PA and weight self-monitoring | Fogg Behaviour Model | Rule-based (whether notification clicked and data logged) | Notifications prompted daily/weekly logging of weight, diet, and PA, plus self-ratings twice weekly. They were triggered at scheduled times, when data were missing, or during PA transitions (context group). Each notification was tracked with three flags: sent (data already logged), clicked (opened within 60 min), and completed (data logged within 60 min). |
| Sweetch [51] | Passive (calendar, location, digital phenotype, weight scale) | Feedback only via application messages  Personalized push notifications that guide users into achieving recommended activity, weight reduction, and diet goals | PA, weight self-monitoring, diet | Transtheoretical Mode | ML (learns what types of messages result in better compliance for a specific user a specific context) | App translates phone and scale data into insights on life habits and learns which messages improve compliance by context (time, day, location, message history). It tracks daily/weekly activity progress, visualizes weight change and goals, and delivers personalized push notifications with actionable recommendations. |
| Fit4Life *(conceptual)* [52] | Passive (data recorder, earpiece, Thinsert, heart rate monitor, metabolic lancet) | Prompt and feedback via audio feedback    Audio feedback, prompts social support via wearables, posts progress updates on social media | BMI, diet, PA | Not stated | Rule-based | Data from wearable sensors generates audio feedback, prompts social support, and posts progress updates to social media for encouragement. Fit4Life is a fictional prototype to explore ethical implications |
| ThinkSlim  [53] | Active (EMA –random [input at random time] vs event sampling [input prior to eating]) | Prompt and feedback via application message  Adaptive feedback via app notifications | Unhealthy eating events | Not stated | ML (classification decision trees and hierarchical agglomerative clustering) | Study I data were used to derive 65 rules and cluster participants by eating behavior (6 clusters formed). In Study II, participants were assigned to groups and received tailored feedback: each new entry was checked against their rule set (via decision tree), triggering warnings when matched. Individual tailoring included statistically relevant personal rules identified during the first week. |
| Android app + Fitbit One [57] | Passive (step count) | Prompts, feedback and educational information via text messages  Tailored SMS messages, weekly exercise education links and daily step reports | Sedentary time | Not stated | Rule-based (inactivity threshold triggers tailored SMS reminders) | If <15 steps/hr (no blackout -user specified not to send notifications at this time period) → SMS prompt with PA break suggestions  If user no sync >1h/24h → sync reminder  Weekly exercise education materials and daily step summary also sent to user |
| 10sFork  [54] | Passive (fork sensors/actuators that provide real time vibrotactile feedback on eating rate) | Feedback only via vibrotactile feedback  Vibrotactile feedback (fork vibrates if bites <10s apart); online dashboard gave retrospective visual feedback (only in 1 group) | Bite rate, success ratio (bite >10s), BMI | Not stated | Rule-based (<10s = vibration + red light) | Fork prongs detect bite via short circuit → timestamp stored → vibrotactile + red light feedback if <10s between bites → logs bite count/intervals Participants with access to online dashboard = retrospective visual feedback (meal patterns and over-speed ratio of past meals) |
| Android app + Wrist-Worn Sensor [58] | Passive (bites taken from wrist accelerometer) | Prompt and feedback via audio feedback and text messages  Real-time feedback (bite count, rate, speed); audio and text messages (if speed> 5 bites/min) | Eating rate | Not stated | Rule-based (peaks in acceleration data 🡪 bite detection; >5 bites/min = warning) | Wrist accelerometer detects bites (smoothed peaks) → data sent via Bluetooth → real-time feedback on bites, rate, speed → audio/text warning if >5 bites/min. |
| JITAI SMS messages [59][57] | Passive (image capturing sensors) | Prompt and feedback via text messages  SMS messages (energy intake and eating rate, numeric vs non-numeric messages) | Amount of food intake and speed | Not stated | Rule-based | Participants assigned into two JITAIs (Energy Intake + Eating Rate) → system tracks daily intake & eating speed → sends numeric (calorie % or timing goals) or non-numeric (pause/reassess prompts) messages → each participant gets 8 per system (4 numeric + 4 non-numeric) → total 16 random messages → drawn from 48 variations. |
| Automated Diet Monitoring Intelligent Personal Assistant (ADM-IPA) [55] | Passive (chewing and swallowing sounds captured by Bluetooth headsets) | Prompt and feedback via audio feedback  Automated detection of eating behaviors (overeating, snacking, skipping meals, irregular timing) and real-time feedback and corrective prompts after eating | Unhealthy eating frequency, timing, and portion size | Fogg Behaviour Model, Transtheoretical Model | ML (deep learning – deep belief network) | Participants wore Bluetooth headsets → chewing and swallowing sounds captured → audio features extracted on smartphone → features sent to cloud ADM backend → deep learning algorithms classified eating actions → system-defined eating events → IPA chatbot delivered just-in-time voice reminders and prompts |

API: Application Programming Interface.

BCT: behavior change technique.

EMA: ecological momentary assessment.

JITAI: just-in-time adaptive intervention.

NR: not reported.

PA: physical activity
